# Solute Carrier Family 37 Member 2 (SLC37A2) Negatively Regulates Murine Macrophage Inflammation by Controlling Glycolysis

**DOI:** 10.1016/j.isci.2020.101125

**Published:** 2020-05-04

**Authors:** Zhan Wang, Qingxia Zhao, Yan Nie, Yi Yu, Biswapriya B. Misra, Manal Zabalawi, Jeff W. Chou, Chia-Chi C. Key, Anthony J. Molina, Matthew A. Quinn, Michael B. Fessler, John S. Parks, Charles E. McCall, Xuewei Zhu

**Affiliations:** 1 Department of Internal Medicine, Section of Molecular Medicine, Wake Forest School of Medicine, Winston-Salem, NC 27157, USA; 2 Division of Public Health Sciences, Wake Forest School of Medicine, Winston-Salem, NC 27157, USA; 3 Department of Microbiology and Immunology, Wake Forest School of Medicine, Winston-Salem, NC 27157, USA; 4 Department of Pathology, Wake Forest School of Medicine, Winston-Salem, NC 27157, USA; 5 Department of Pediatrics, Ruijin Hospital, Shanghai Jiaotong University School of Medicine, Shanghai 200025, China; 6 Department of Internal Medicine, Section on Gerontology and Geriatric Medicine, Wake Forest School of Medicine, Winston-Salem, NC 27157, USA; 7 Immunity, Inflammation and Disease Laboratory, National Institute of Environmental Health Sciences, NIH, Research Triangle Park, NC 27709, USA

**Keywords:** Cell Biology, Functional Aspects of Cell Biology, Immunology, Metabolism

## Abstract

Increased flux of glucose through glycolysis is a hallmark of inflammatory macrophages and is essential for optimal effector functions. Solute carrier (SLC) 37A2 is an endoplasmic reticulum-anchored phosphate-linked glucose-6-phosphate transporter that is highly expressed in macrophages and neutrophils. We demonstrate that SLC37A2 plays a pivotal role in murine macrophage inflammatory activation and cellular metabolic rewiring. Toll-like receptor (TLR) 4 stimulation by lipopolysaccharide (LPS) rapidly increases macrophage SLC37A2 protein expression. SLC37A2 deletion reprograms macrophages to a hyper-glycolytic process and accelerates LPS-induced inflammatory cytokine production, which partially depends on nicotinamide adenine dinucleotide (NAD^+^) biosynthesis. Blockade of glycolysis normalizes the differential expression of pro-inflammatory cytokines between control and SLC37A2 deficient macrophages. Conversely, overexpression of SLC37A2 lowers macrophage glycolysis and significantly reduces LPS-induced pro-inflammatory cytokine expression. In conclusion, our study suggests that SLC37A2 dampens murine macrophage inflammation by down-regulating glycolytic reprogramming as a part of macrophage negative feedback system to curtail acute innate activation.

## Highlights

• LPS treatment rapidly elevates macrophage SLC37A2 protein expression
• SLC37A2 dampens early glycolytic reprogramming in acute macrophage inflammation
• SLC37A2 suppresses macrophage cell-surface and endosomal TLR activation
• SLC37A2 attenuates macrophage cellular ROS production

## Graphical Abstract

**Figure:**
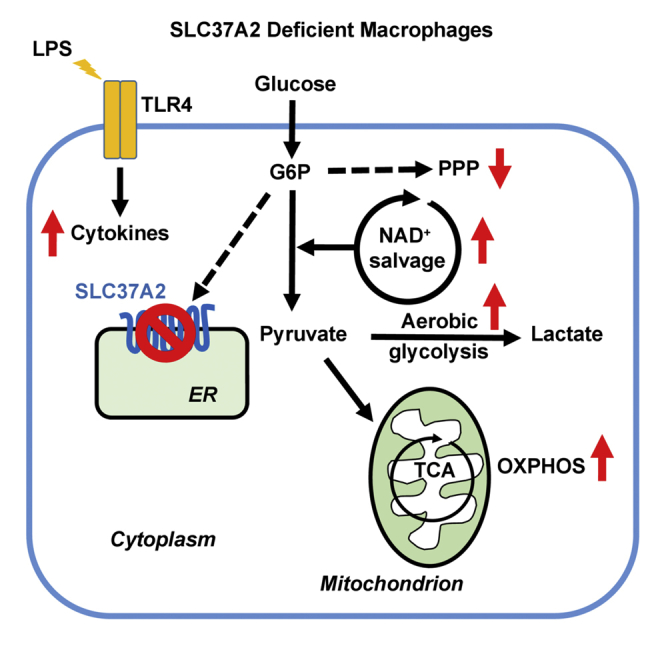

## Introduction

Innate immune cells, such as circulating blood monocytes and tissue-resident macrophages, serve as the first line of immune defense against invading pathogens. Macrophages are one of the key drivers in the pathogenesis of chronic metabolic diseases, including type 2 diabetes and atherosclerosis. Understanding the mechanism underpinning macrophage activation is critical for designing therapeutic targets for inflammatory diseases. Macrophages exhibit a heterogeneous phenotype that has been classified as classical or alternative activation, depending on the micro-environmental cues (Murray et al., 2014). Classical activation of macrophages by interferon-gamma and lipopolysaccharide (LPS), a Toll-like receptor (TLR) 4 agonist, or LPS alone rapidly triggers immune effector functions such as cytokine secretion, phagocytosis, and chemotaxis, to support an antimicrobial program.

In response to inflammatory stimuli, monocytes and macrophages sequentially reconfigure metabolism and bioenergetics (Zhu et al., 2019), which not only provides energy and substrates for cell survival but also regulates or instructs immune effector functions. Growing evidence indicates that classical activation of macrophages rewires cellular metabolism toward aerobic glycolysis to meet the energy demands of inflammatory cytokine, lipid, protein, and nucleotide biosynthesis (Kelly and O'Neill, 2015, O'Neill and Pearce, 2016, Pearce and Pearce, 2013, Torres et al., 2016). Classical activation rapidly induces glucose uptake, initiates glycolytic reprogramming, and concomitantly suppresses fatty acid uptake and oxidation (Vats et al., 2006, Rodriguez-Prados et al., 2010) in macrophages. To date, different mechanisms of glucose metabolism rewiring in inflammatory myeloid cells driven by classical/TLR activation have been described, for example: (1) increased plasma membrane glucose transporter 1 (GLUT1) expression, which increases glucose uptake and subsequent glycolysis and pentose phosphate pathway (PPP) flux (Fukuzumi et al., 1996, Freemerman et al., 2014, Freemerman et al., 2019, Nishizawa et al., 2014); (2) increased hexokinase (HK) activity that converts glucose to glucose-6-phosphate (G6P) (Perrin-Cocon et al., 2018); (3) induced phosphofructokinase (PFK)2 isoform shift from low-activity liver type PFK2 (PFKL) to the more active ubiquitous PFK2 (PFKFB3), which keeps high level of fructose-2,6-bisphosphate to support glycolysis (Minchenko et al., 2002, Obach et al., 2004); (4) increased pyruvate kinase (PK), muscle (PKM)2-dependent glycolysis (Xie et al., 2016, Palsson-McDermott et al., 2015); (5) down-regulated carbohydrate kinase-like protein (CARKL), which regulates glucose flux through the non-oxidative arm of the PPP (Haschemi et al., 2012); and very recently (6) activated nicotinamide adenine dinucleotide (NAD^+^) salvage pathway, which is required for the maintenance of NAD^+^ pools for glyceraldehyde-3-phosphate dehydrogenase (GAPDH) activity and glycolytic activation (Cameron et al., 2019). Together, these findings suggest that increased flux of glucose through glycolysis and the PPP is a crucial feature of inflammatory myeloid cells and is required for optimal inflammatory activation of these cells. Moreover, emerging mechanisms of glycolytic reprogramming suggest that activated macrophage glucose and energy metabolism is not fully characterized.

The solute carrier family 37 (SLC37) consists of four sugar-phosphate exchangers, A1, A2, A3, and A4, which are anchored in the endoplasmic reticulum (ER) membrane (Chou et al., 2013). SLC37A4 (also known as the G6P transporter [G6PT]) is the best-characterized family member and functions as a phosphate-linked G6P antiporter. Deficiencies in G6PT cause glycogen storage disease type Ib, characterized by hypoglycemia and neutropenia and neutrophil dysfunction (Chou et al., 2010). In the liver, kidney, and intestine, SLC37A4 (G6PT) couples functionally with glucose-6-phosphatase-α (G6Pase-α) to maintain interprandial blood glucose homeostasis, whereas in neutrophils it couples functionally with G6Pase-β to maintain neutrophil energy homeostasis by recycling of ER glucose to the cytoplasm (Jun et al., 2010, Jun et al., 2012, Kim et al., 2008). SLC37A2 is also a Pi-linked G6P antiporter but does not functionally couple with G6Pase-α or G6Pase-β (Pan et al., 2011). Interestingly, of the four SLC37A members, SLC37A2 displays the highest level of transcript abundance in neutrophils and macrophages (Pan et al., 2011, Kim et al., 2007, Chou et al., 2013), indicating that SLC37A2 may play an essential role in regulating innate immune function. However, whether, how, and to what extent SLC37A2 controls macrophage glucose metabolism and inflammation is unknown.

In the current study, using high-throughput transcriptomic and metabolomic approaches, we demonstrated that SLC37A2 is pivotal in the regulation of murine macrophage inflammatory activation and cellular metabolic rewiring. As LPS rapidly up-regulates SLC37A2 protein expression, our study suggests that, by fine-tuning glycolytic reprogramming, SLC37A2 functions as an early repressor to dampen macrophage inflammatory activation and to promote resolution of inflammation during acute inflammatory activation.

## Results

### Deletion of SLC37A2 Promotes Macrophage Pro-Inflammatory Activation

**Figure 1:**
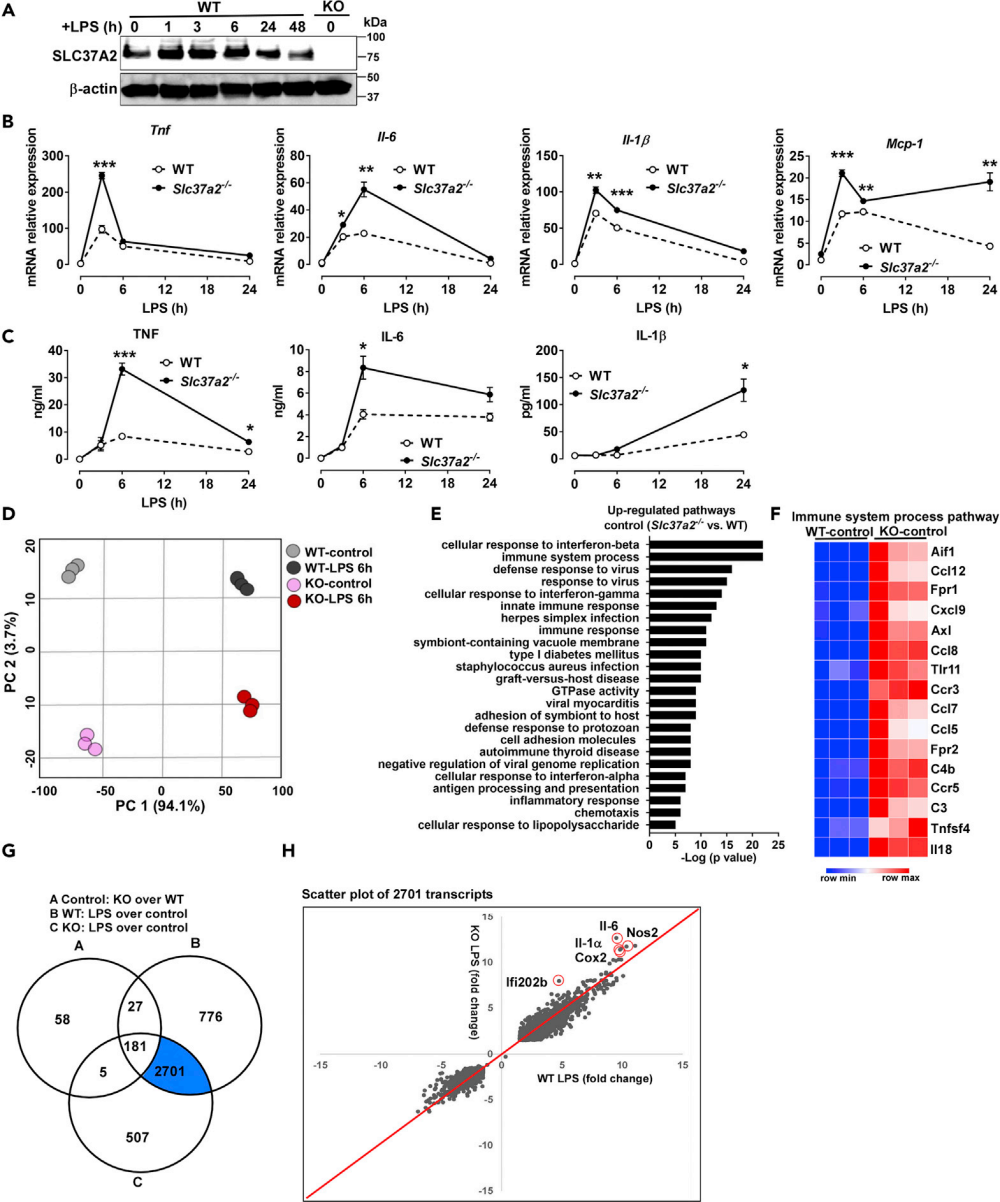
Deletion of SLC37A2 Promotes Macrophage Pro-Inflammatory Activation (A) SLC37A2 protein expression in WT bone marrow-derived macrophages (BMDMs) treated with 100 ng/mL LPS for 0–48 h, measured by western blotting. BMDMs from *Slc37a2*^*−/−*^ mice were used as a negative control. (B and C) Cytokine mRNA and protein expression in WT and *Slc37a2*^*−/−*^ BMDMs stimulated with 100 ng/mL LPS for 0–24 h, measured by qPCR and ELISA, respectively. (D) Principal component analysis of the normalized RNA-seq abundance data (transcripts per million) from BMDMs treated with or without 100 ng/mL LPS for 6 h. (E) The top up-regulated pathways in *Slc37a2*^*−/−*^ versus WT BMDMs in the absence of LPS stimulation, generated from pathway enrichment analysis. (F) Heatmap of genes in the top most enriched pathway “immune system process pathway” shown in (E). (G) A three-way Venn diagram displaying the numbers of unique and shared transcripts with log2 fold change of expression over 1.5 in *Slc37a2*^*−/−*^ versus WT macrophages at the baseline, WT macrophages treated with LPS versus control, and *Slc37a2*^*−/−*^ macrophages treated with LPS versus control. (H) Scatterplot of LPS-induced transcripts. Each dot represents a gene. The x and y axis represent the LPS-induced gene log2 fold change in WT and *Slc37a2*^*−/−*^ macrophages, respectively. KO, *Slc37a2*^*−/−*^. Data are representative of three independent experiments with three samples per group (mean ± SEM). ∗p < 0.05, ∗∗p < 0.01, ∗∗∗p < 0.001, unpaired, two-tailed Student's t test.

SLC37A2 is highly expressed in macrophages, and its expression is significantly up-regulated in inflamed high-fat diet-fed adipose tissues (Pan et al., 2011, Kim et al., 2007), suggesting a link between SLC37A2 and inflammation. To investigate the possible role of SLC37A2 in macrophage inflammation, we first measured SLC37A2 protein expression in macrophages in response to LPS stimulation. Interestingly, LPS rapidly increased SLC37A2 protein level in wild-type (WT) bone marrow-derived macrophages (BMDMs) at 1 h. SLC37A2 protein level remained high at 3 and 6 h and declined at 24 and 48 h of LPS stimulation. As a negative control, SLC37A2 protein was undetectable in BMDMs from *Slc37a2* knockout (KO, *Slc37a2*^*−/−*^) mice (Figure 1A).

To assess the role of SLC37A2 in macrophage activation, we first examined cytokine production in BMDMs from WT and *Slc37a2*^*−/−*^ mice treated with 100 ng/mL LPS (TLR4 agonist) during a 24-h period. Relative to WT control, SLC37A2-deficient BMDMs were more prone to LPS-induced inflammatory activation, as shown by significantly increased transcripts of pro-inflammatory cytokines (*Tnf*, *Il-6*, *Il-1β*, and *Mcp-1*) (Figure 1B). Consistent with mRNA expression, pro-inflammatory cytokine (TNF, IL-6, and IL-1β) secretion from *Slc37a2*^*−/−*^ BMDMs in response to LPS stimulation was significantly higher than in WT (Figure 1C), suggesting that SLC37A2 deficiency enhances macrophage TLR4 activation. Similar results were obtained when we used thioglycollate-elicited peritoneal macrophages from WT and *Slc37a2*^*−/−*^ mice (Figure S1) or siRNA-silenced peritoneal macrophages from WT mice stimulated with LPS (Figures S2A and S2B). SLC37A2-deficient macrophages also produced more IL-6 and TNF in response to Pam3CSK4 (cell-surface TLR2), poly (I:C) (endosomal TLR3), loxoribine (endosomal TLR7), and CpG (endosomal TLR9), respectively (Figures S3A–S3D and S2C), suggesting that SLC37A2 has a broad inhibitory effect on TLR pathways. In addition to TLRs, we also examined the inflammatory responses of *Slc37a2*^*−/−*^ versus WT macrophages in response to the cytosolic NOD-, LRR-, and pyrin domain-containing protein 3 (NLRP3) inflammasome activation. Activation of the NLRP3 inflammasome requires two signals: the first “priming” signal, which enhances the transcription of pro-IL-1β and NLRP3 via TLR activation, and the second “activation” signal, which promotes the assembly of the inflammasome complex and cleavage of caspase-1 (Mangan et al., 2018, Swanson et al., 2019). We found that, compared with WT, LPS-primed *Slc37a2*^*−/−*^ BMDMs secreted a higher level of IL-1β but had no difference in caspase-1 cleavage in response to ATP, a specific stimulus of the NLRP3 inflammasome (Figures S3E and S3F), suggesting that SLC37A2 deficiency promotes ATP-induced IL-1β secretion primarily by up-regulating TLR4 activation. Taken together, our results indicate that SLC37A2 represses TLR activation and negatively regulates macrophage inflammation.

To get a more in-depth and system-wide insight into the regulatory roles of SLC37A2 in macrophage inflammation, we performed transcriptomic analysis via RNA sequencing (RNA-seq). An unsupervised principal component analysis (PCA) revealed a clear separation based on genotype and treatment, i.e., LPS stimulation (Figure 1D), where the first two principal components (PCs 1 and 2) alone cumulatively explained 97.7% of the variance in the data. Further pathway enrichment analysis revealed that the up-regulated pathways in the resting *Slc37a2*^*−/−*^ versus WT macrophages were enriched for “immune system process,” “cellular response to interferon-β," “innate immune response,” “cell adhesion molecules,” and “chemotaxis” (Figure 1E). The transcripts identified in the most significantly enriched pathway (“immune system process” pathway) included several differentially regulated chemokines (*Ccl12*, *Cxcl9*, *Ccl8*, *Ccl7*, and others), chemokine receptors (*Ccr3*, *Ccr5*), and cytokines (*Tnfsf4*, *Il-18*) in resting *Slc37a2*^*−/−*^ versus WT macrophages (Figure 1F). The up-regulated cellular response to interferon-β in SLC37A2-deficient macrophages was confirmed by qPCR, as shown by increased expression of canonical type I interferon-response genes, including *Mx1*, *Mx2*, and *Interferon-stimulated gene (Isg)15* in resting *Slc37a2*^*−/−*^ versus WT macrophages (Figure S4). Interestingly, SLC37A2 deficiency only had a mild effect on interferon-stimulated gene expression in macrophages when treated with murine interferon-β (Figure S4). Together, these results indicate that SLC37A2 deletion creates an inflammatory primed condition even in the absence of an exogenous inflammatory stimulus. Further analysis of RNA-seq data revealed the number of transcripts with log2 fold change of expression over 1.5 in *Slc37a2*^*−/−*^ versus WT macrophages at the baseline (271 transcripts), WT macrophages treated with LPS versus control (3,685 transcripts), and KO macrophages treated with LPS versus control (3,394 transcripts) (Figure 1G). In a scatterplot of LPS-regulated 2,701 transcripts (highlighted in blue in Figure 1G), *Il-6*, *Nos2*, *Cox2*, *Il-1α,* and *Ifi202b* were highly significantly increased in *Slc37a2*^*−/−*^ versus WT macrophages (Figure 1H). Collectively, these data indicate that SLC37A2 functions as a repressor of macrophage activation. In the absence of SLC37A2, macrophages are more prone to inflammatory activation. Hence, the rapid up-regulation of SLC37A2 by TLR4 activation (Figure 1A) is likely critical for macrophages to suppress hyper-inflammatory activation during the early phase of inflammation.

### Activation of MEK/Erk1/2 Pathway Is Involved in the Hyper-Inflammation of *Slc37a2*^*−/−*^ Macrophages

Nuclear factor (NF)-κB, mitogen-activated protein kinase (MAPK), and PI3K/Akt pathways are three major signaling pathways involved in TLR4-mediated inflammatory activation in macrophages (Kawai and Akira, 2010, Kawai and Akira, 2011, Dauphinee and Karsan, 2006). The mammalian target of rapamycin (mTOR) is a central integrator of cellular metabolism, and its signaling also regulates innate and adaptive immunity (Jones and Pearce, 2017). PI3K can directly activate mTOR complex (mTORC) 2, which then phosphorylates Akt1 at Ser473 (p-Akt (S473)), thus promoting Akt-mediated activation of HK2 and PFK. PI3K also can indirectly activate mTORC1 via phosphorylating Akt1 at Thr308 (p-Akt (T308)). Active mTORC1 phosphorylates S6K and 4E-BP, promoting lipid and nucleic acid synthesis and activating hypoxia-inducible factor-1 alpha (HIF-1α) to support cell growth and effector functions. Although it is context dependent, in general, mTORC1 activity is anti-inflammatory, whereas mTORC2 is pro-inflammatory (Jones and Pearce, 2017).

**Figure 2:**
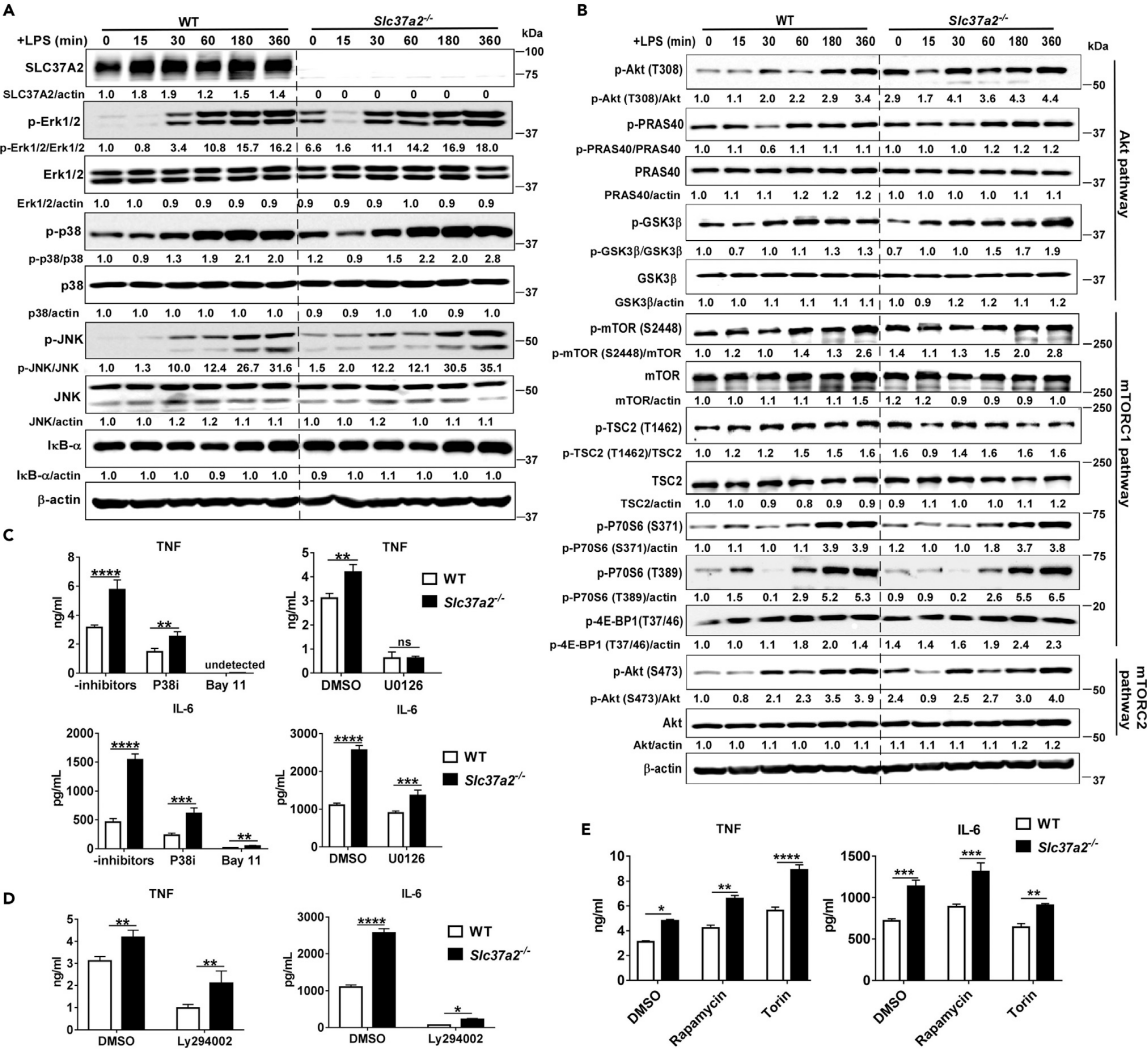
MEK/Erk1/2 Signaling Is Involved in SLC37A2-Mediated Inflammation (A and B) BMDMs from WT and *Slc37a2*^*−/−*^ mice were treated with 100 ng/mL LPS for 0–6 h. Cell lysates were used for immunoblotting signal molecules in MAPK and NF-κB pathways (A) and PI3K/Akt1, mTORC1, and mTORC2 pathways (B). The intensity of each target protein band was normalized to its total (unphosphorylated) form or β-actin. The intensity of WT macrophage protein band at 0 h was set to 1; the intensity of each protein band relative to the WT control macrophages at 0 h is then calculated. The results are representative of two independent experiments. The average of the densitometry of the two experiments was shown under each protein band. (C–E) Secretion of cytokines from WT and *Slc37a2*^*−/−*^ BMDMs pretreated with IκBα inhibitor Bay 11-7082 (Bay11, 5 μM), p38 MAPK inhibitor III (p38i, 2 μM), MEK/Erk1/2 inhibitor U0126 (25 μM), PI3K inhibitor Ly294002 (20 μM), mTOR inhibitors rapamycin (50 nM), and Torin 1 (100 nM), followed by 100 ng/mL LPS for 6 h in the presence of inhibitors. Data are representative of two independent experiments with 4 samples per group (mean ± SEM). ∗p < 0.05, ∗∗p < 0.01, ∗∗∗p < 0.001, ∗∗∗∗p < 0.001, unpaired, two-tailed Student's t test.

To assess whether and to what extent these signaling pathways contribute to hyper-inflammation in *Slc37a2*^*−/−*^ macrophages, we immunoblotted the key signaling molecules in each pathway and then examined their functional contributions to hyper-inflammation of *Slc37a2*^*−/−*^ macrophages using pharmacological inhibitors of each pathway. Among the signaling pathways we examined, the MAPK/MEK/Erk1/2 pathway, in particular, showed increased activation in *Slc37a2*^*−/−*^ macrophages, as demonstrated by a striking increase in phosphorylation of Erk1/2 at baseline (Figure 2A). SLC37A2 deficiency showed a minor effect on the activation of other signaling pathways (Figures 2A and 2B). Next, we pretreated WT and *Slc37a2*^*−/−*^ macrophages with inhibitors of the NF-κB (IκBα phosphorylation inhibitor Bay11-7082), MAPK (p38 inhibitor III and MEK/Erk1/2 inhibitor U0126), PI3K (Ly294002), and mTORC1/2 (rapamycin and Torin-1) pathways before stimulating cells with LPS. Blockade of NF-κB, PI3K, P38, or MEK activation significantly lowered cytokine (IL-6 and TNF) expression in both WT and *Slc37a2*^*−/−*^ macrophages (Figures 2C–2D). In contrast, inhibition of mTOR by rapamycin significantly elevated cytokine (IL-6 and TNF) expression in both genotypic macrophages (Figure 2E). Inhibition of mTOR by Torin significantly elevated TNF but slightly reduced IL-6 secretion in both genotypes, demonstrating a cytokine-specific effect of mTOR inhibition by Torin on cytokine production (Figure 2E). However, among the inhibitors we tested, only MEK/Erk1/2 inhibitor U0126 normalized the differential expression of TNF, and partially normalized the difference in IL-6 between the two genotypes (Figure 2C). In summary, our results indicate that among the signaling pathways investigated, only the MEK/Erk1/2 pathway seems to play a role in the elevated production of TNF in *Slc37a2*^*−/−*^ macrophages.

Additionally, given SLC37A2 is an ER-anchored G6P antiporter, we speculated that SLC37A2 deletion might impair ER function and/or metabolic homeostasis, perhaps leading to ER stress. However, there is no apparent ER stress in SLC37A2-deficient macrophages at the basal level or after 6 h of LPS exposure, as the expression level of the ER stress marker C/E homologous protein (CHOP) was comparable between genotypes (Figure S5). The role of SLC37A2 in ER metabolism and function is unknown and will be investigated in our future studies.

### SLC37A2 Deletion Alters Macrophage Metabolism at Both Transcriptional and Metabolic Levels

**Figure 3:**
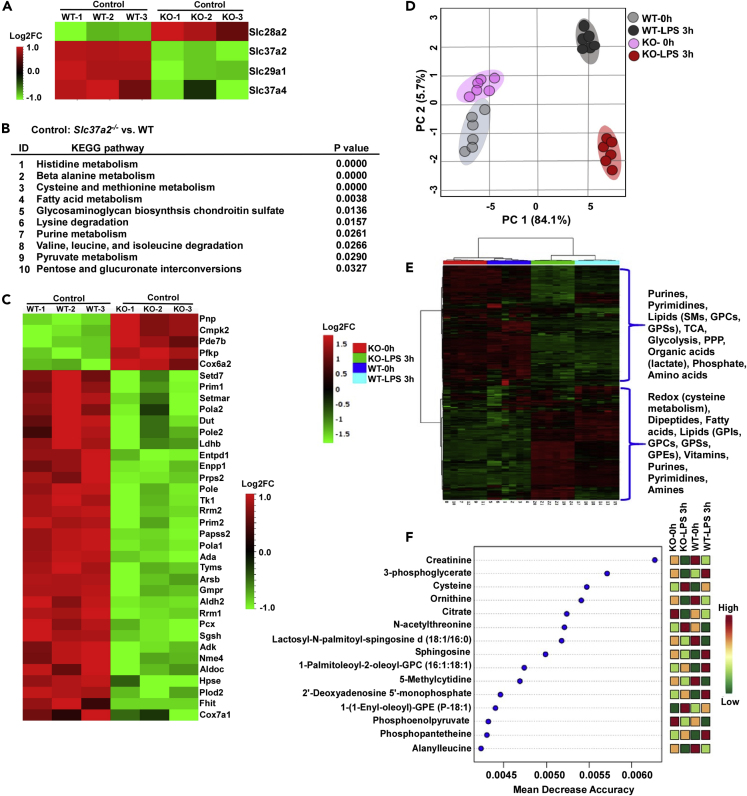
SLC37A2 Deletion Broadly Rewires Macrophage Metabolism at Both Transcriptional and Metabolic Levels (A) Heatmap of genes in the pathway of carbohydrate derivative transporters from resting WT and *Slc37a2*^*−/−*^ BMDMs, assessed by RNA-seq (three biologically independent samples per group). (B) The enriched metabolic KEGG pathways in resting *Slc37a2*^*−/−*^ versus WT BMDMs are shown and ranked according to p values, assessed by RNA-seq. (C) Heatmap of genes under the category of metabolism in resting WT and versus *Slc37a2*^*−/−*^ BMDMs, determined by RNA-seq. (D) Score plots of principal component analysis of the untargeted metabolomics data collected using WT and *Slc37a2*^*−/−*^ BMDMs treated with 100 ng/mL LPS for 0 or 3 h (n = 6 per group). (E) Heatmap displaying hierarchical clustering analysis of metabolites in WT and *Slc37a2*^*−/−*^ BMDMs treated with 100 ng/mL LPS for 0 or 3 h. (F) Results of random forest analysis showing mean decrease accuracies of the top 15 metabolites altered in WT and *Slc37a2*^*−/−*^ BMDMs treated with 100 ng/mL LPS for 0 or 3 h. KO, Slc37a2^−/−^.

As SLC37A2 is a reported phosphate-linked G6P transporter, we then asked if the deletion of SLC37A2 in macrophages would alter carbohydrate and/or other types of cellular metabolism (i.e., energy) at the transcriptional and metabolic levels. As shown in our RNA-seq data, *Slc37a2* was the top down-regulated transcript in *Slc37a2*^*−/−*^ versus WT macrophages in the “carbohydrate derivative transport” pathway (Figure 3A). The deletion of SLC37A2 did not enhance *Slc37a4* in KO macrophages. Interestingly, we observed increased *Slc28a2* but decreased *Slc29a1*, both of which are nucleoside transporter proteins, suggesting that the deletion of SLC37A2 possibly alters nucleotide metabolism (Figure 3A). Notably, the pathways of “cysteine and methionine metabolism,” “fatty acid metabolism,” “purine metabolism,” “pyruvate metabolism,” and “pentose and glucuronate interconversions” significantly differed at the transcriptional levels between WT and *Slc37a2*^*−/−*^ macrophages, suggesting that SLC37A2 is crucial in the maintenance of metabolic homeostasis in macrophages, at least at the gene expression levels (Figure 3B). The differentially regulated transcripts in resting *Slc37a2*^*−/−*^ versus WT macrophages, which have functional annotations associated with “metabolism” from KEGG pathway analysis, are displayed in a heatmap generated using hierarchical clustering analysis (Figure 3C). Among them, the top up-regulated transcripts in *Slc37a2*^*−/−*^ versus WT macrophages were purine nucleoside phosphorylase (*Pnp*), cytidine/uridine monophosphate kinase 2 (*Cmpk2*), and phosphodiesterase 7B (*Pde7b*), which regulate nucleotide homeostasis, as well as phosphofructokinase, platelet type (*Pfkp*, one of the most important regulatory enzymes of glycolysis), and cytochrome *c* oxidase subunit 6A2 (*Cox6a2*, also known as Complex IV).

In addition to the transcriptomic analysis, we also performed untargeted metabolomics analysis using WT and *Slc37a2*^*−/−*^ BMDMs treated with or without LPS for 3 h. A total of 508 confidently identified metabolites and lipids were quantified in macrophages. A PCA (Figure 3D) score plot successfully segregated the groups based on LPS treatment and genotypes on the first two PCs 1 and 2, explaining 89.8% of the variance, suggesting distinct metabolic effects of SLC37A2 deficiency in resting and LPS-stimulated macrophages. A heatmap (Figure 3E) display of hierarchical cluster analysis (HCA) of the metabolites (including lipids, nucleotides, glycolytic intermediates, redox metabolites, and TCA cycle metabolites) by LPS treatment, with clear sub-clustering of samples by genotypes, suggests that SLC37A2 deletion has a broad impact on macrophage metabolism. A random forest analysis derived from a supervised multivariate analysis yielded the mean decreased accuracies (MDAs) for the “top 15” metabolites that strongly contributed to the binning of individual samples into groups (Figure 3F). These top 15 metabolites included metabolites belonging to glycolysis (3-phosphoglycerate and phosphoenolpyruvate [PEP]), TCA cycle metabolism (citrate), redox metabolism (cysteine), lipid metabolism (lactosyl-N-palmitoyl-sphingosine D [18:1/16:0], sphingosine, 1-(1-enyl-oleoyl)-GPE [p-18:1]), nucleotide metabolism (5-methylcytidine), and amino acid metabolism (including ornithine, N-acetyl threonine, and alanylleucine). Taken together, these results indicate that the deletion of SLC37A2 broadly influences cellular glucose, redox, lipid, amino acid, and nucleic acid metabolism at both transcription and metabolism levels and SLC37A2 is a vital transporter controlling cellular metabolic homeostasis.

### SLC37A2 Deletion Accelerates Glycolysis and Enhances Mitochondrial Oxidative Phosphorylation in Resting Macrophages

**Figure 4:**
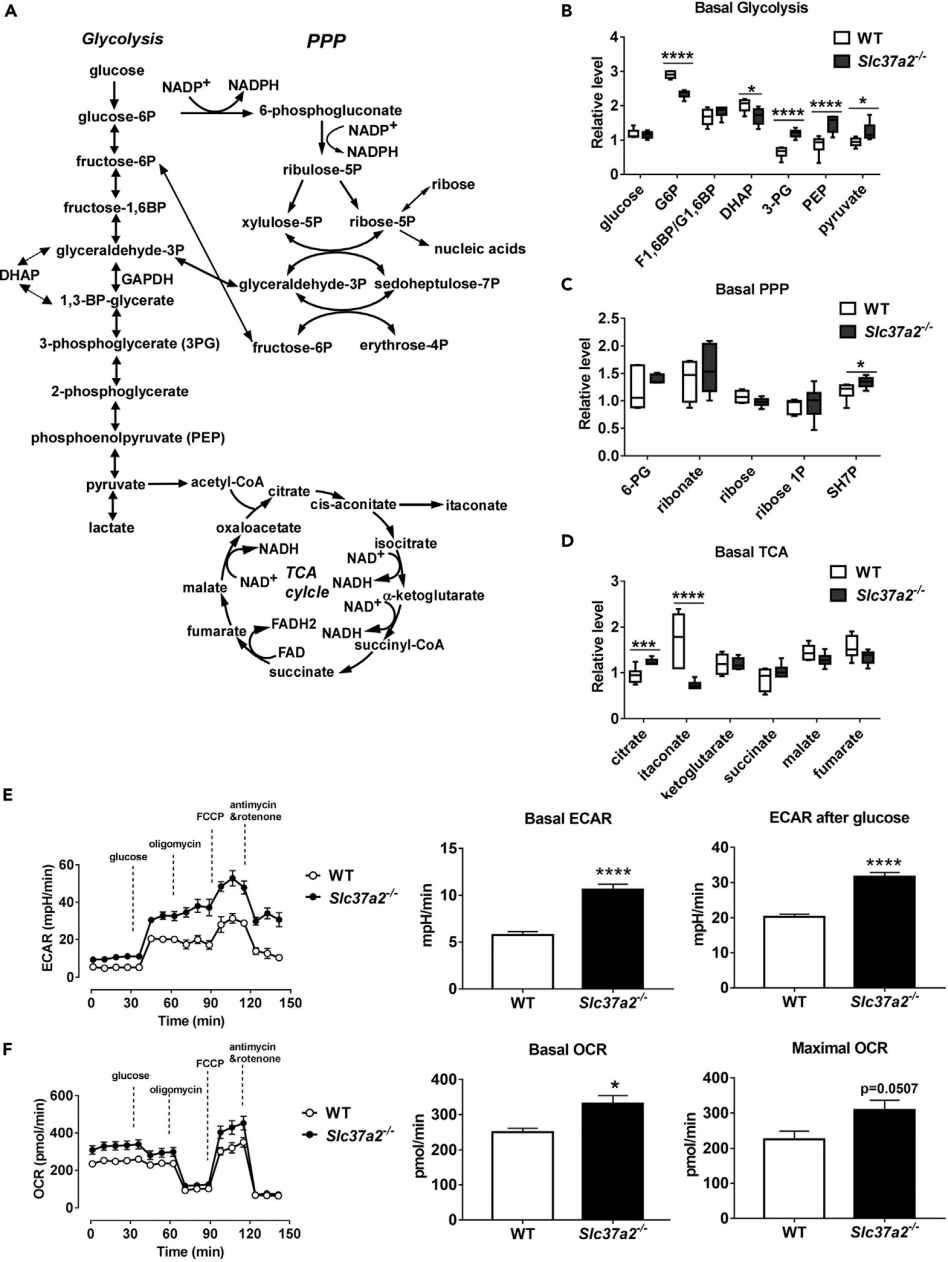
SLC37A2 Deletion Accelerates Basal Glycolysis and Mitochondrial Oxidative Phosphorylation (A) Diagram of glycolysis, pentose phosphate pathway (PPP), and TCA cycle. (B–D) Basal levels of metabolites in resting WT and *Slc37a2*^*−/−*^ BMDMs in the pathways of glycolysis, PPP, and TCA, respectively. (E) Seahorse analysis of basal extracellular acidification rates (ECAR) and ECAR after glucose injection in resting WT and *Slc37a2*^*−/−*^ BMDMs. (F) Seahorse analysis of basal and maximal oxygen consumption rate (OCR) in resting WT and *Slc37a2*^*−/−*^ BMDMs. Data are represented as mean ± SEM (n = 6 per group). ∗p < 0.05, ∗∗∗p < 0.001, ∗∗∗∗p < 0.001, unpaired, two-tailed Student's t test.

It is well established that pro-inflammatory macrophage activation triggers a Warburg-like shift toward aerobic glycolysis (Kelly and O'Neill, 2015). As SLC37A2 deletion promotes hyper-proinflammation and globally affects macrophage metabolism, we next examined the changes in *Slc37a2*^*−/−*^ versus WT macrophages in the pathways of glycolysis, PPP, and TCA cycle (Figure 4A). We observed a significant decrease in G6P and dihydroxyacetone phosphate (DHAP) in resting *Slc37a2*^*−/−*^ versus WT macrophages. Interestingly, the levels of 3-phosphoglycerate, PEP, and pyruvate, which are all glycolytic intermediates downstream of GAPDH, a rate-limiting step in aerobic glycolysis (Shestov et al., 2014), were significantly higher in *Slc37a2*^*−/−*^ versus WT macrophages (Figure 4B), suggesting that SLC37A2-deficient macrophages accelerate GAPDH-mediated glycolysis in the absence of TLR stimulation.

In addition to increased aerobic glycolysis, macrophages also show increased flux through the PPP upon pro-inflammatory activation (Haschemi et al., 2012). In our study, in the absence of LPS stimulation, we only observed a significant increase in sedoheptulose-7-phosphate (SH7P) in PPP in *Slc37a2*^*−/−*^ versus WT macrophages, whereas no difference was observed in other PPP intermediates, suggesting that SLC37A2 deletion only mildly increases PPP flux in resting macrophages (Figure 4C).

Recent evidence suggests that the TCA cycle plays a critical role in mediating the anabolic and catabolic reactions of immune metabolism (Ryan et al., 2019, Murphy and O'Neill, 2018). Pro-inflammatory macrophage activation increases the production of citrate (Williams and O'Neill, 2018), likely supporting fatty acid synthesis, and increases the production of succinate, which stabilizes HIF-1α and promotes glycolysis and IL-1β production (Mills et al., 2016, Lampropoulou et al., 2016, Tannahill et al., 2013). Pro-inflammatory macrophage activation also supports the production of the immune-suppressive metabolite itaconate, which promotes immune tolerance (Mills et al., 2016, Mills et al., 2018, Cordes et al., 2016, Lampropoulou et al., 2016, Jha et al., 2015). We found that, at the resting status, *Slc37a2*^*−/−*^ versus WT macrophages showed a significantly increased accumulation of citrate but a significant reduction in itaconate (Figure 4D). Meanwhile, we assessed glycolysis by recording extracellular acidification rate (ECAR) and oxygen consumption rate (OCR) in WT and *Slc37a2*^*−/−*^ macrophages, which were sequentially treated with glucose, oligomycin, fluoro-carbonyl cyanide phenylhydrazone (FCCP), and antimycin and rotenone, by respirometry. Consistent with the metabolomics results, *Slc37a2*^*−/−*^ macrophages showed increased glycolysis (Figure 4E) and enhanced oxidative phosphorylation (OXPHOS) (Figure 4F) at the baseline. Taken together, our results suggest that the deletion of SLC37A2 metabolically primes macrophages to an anabolic pro-inflammatory state.

### SLC37A2 Deletion Accelerates LPS-Induced Glycolysis and Mitochondrial Oxidation

**Figure 5:**
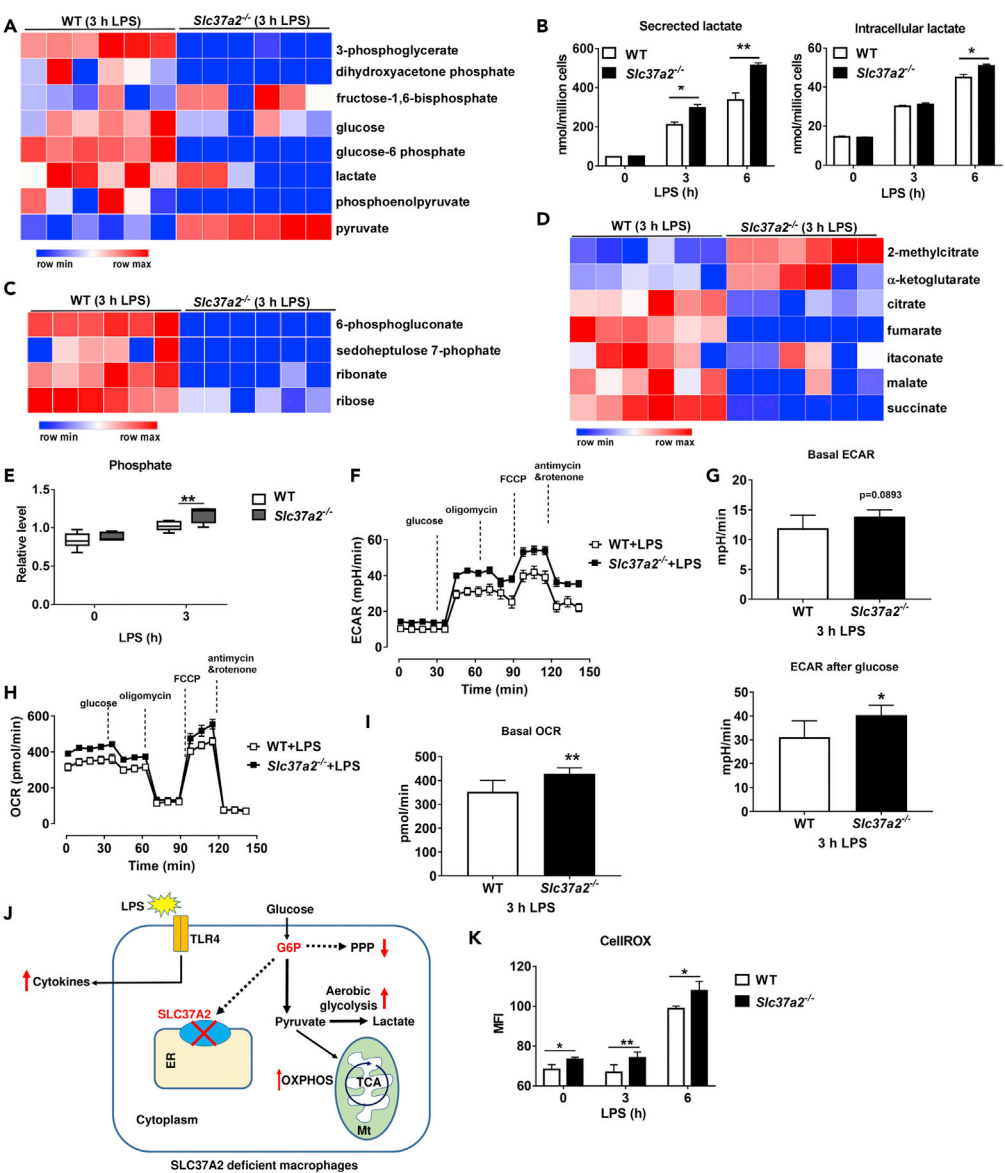
SLC37A2 Deletion Accelerates LPS-Induced Glycolysis and Mitochondrial Oxidation and Promotes Cytosolic Reactive Oxidative Species (ROS) Production (A) Heatmap displays relative levels of glycolytic metabolites in WT and *Slc37a2*^*−/−*^ BMDMs treated with 100 ng/mL LPS for 3 h, assessed by untargeted metabolomics and plotted using row *Z* score. (B) Concentrations of cellular and secreted lactate from WT and *Slc37a2*^*−/−*^ macrophages treated with 100 ng/mL LPS for 0–6 h, measured by a colorimetric kit. (C) Heatmap displays relative levels of pentose phosphate pathway (PPP) metabolites in WT, and *Slc37a2*^*−/−*^ BMDMs treated with 100 ng/mL LPS for 3 h, assessed by unbiased metabolomics and plotted using row *Z* score. (D) Heatmap displays relative levels of TCA cycle metabolites in WT and *Slc37a2*^*−/−*^ BMDMs treated with 100 ng/mL LPS for 3 h, assessed by unbiased metabolomics and plotted using row *Z* score. (E) Phosphate level in WT and *Slc37a2*^*−/−*^ BMDMs treated with or without 100 ng/mL LPS for 3 h, assessed by unbiased metabolomics. (F and G) Seahorse analysis of basal ECAR and ECAR after glucose injection in WT and *Slc37a2*^*−/−*^ BMDMs treated with 100 ng/mL LPS for 3 h. (H and I) Seahorse analysis of basal and maximal OCR in WT and *Slc37a2*^*−/−*^ BMDMs treated with 100 ng/mL LPS for 3 h. (J) Schema for reprogramming of glycolysis, PPP, and TCA cycle metabolism in LPS-stimulated *Slc37a2*^*−/−*^ macrophages. ER, endoplasmic reticulum; Mt, mitochondria; TCA, TCA cycle. (K) BMDMs from WT and *Slc37a2*^*−/−*^ mice were treated with or without 100 ng/mL LPS for 0–6 h. Cells were stained with 5 μM CellROX for 30 min and then analyzed by flow cytometry. Data (mean ± SEM) are representative of at least two independent experiments. ∗p < 0.05, ∗∗p < 0.01, unpaired, two-tailed Student's t test.

Next, we wanted to investigate whether SLC37A2 deletion alters glycolysis, PPP, and TCA cycle in the LPS-stimulated macrophages. Not surprisingly, LPS induced rapid glucose utilization in both WT and *Slc37a2*^*−/−*^ macrophages, as evidenced by decreased glycolytic intermediates, including glucose, G6P, and DHAP, relative to resting macrophages. Interestingly, we observed a significantly higher level of glycolytic end product pyruvate in *Slc37a2*^*−/−*^ versus WT cells post 3 h LPS stimulation, suggesting an increased glycolytic activity (Figure 5A). Secreted lactate, a marker of Warburg-like metabolic effect, measured by an enzymatic assay, was significantly higher at 3 and 6 h of LPS stimulation (Figure 5B) in *Slc37a2*^*−/−*^ versus WT macrophages, confirming the increased glycolysis observed in metabolomics analysis in *Slc37a2*^*−/−*^ macrophages.

Relative to WT, *Slc37a2*^*−/−*^ macrophages showed significantly lower PPP intermediates including 6-phosphogluconate, ribonate, and ribose at 3 h of LPS stimulation (Figure 5C), reflecting generally decreased utilization of glucose upon LPS stimulation for this pathway that generates the anabolic cofactor, the reduced form of nicotinamide adenine dinucleotide (NAD) phosphate (NADPH) and ribose for nucleic acid synthesis. Interestingly, resting *Slc37a2*^*−/−*^ macrophages appeared to have increased nucleotide production, as shown by increased accumulation of nucleotides, including AMP, GMP, CMP, and UDP, but decreased purine and pyrimidine degradation products (xanthine, urate, allantoin, xanthosine, and cytidine). In contrast, 3 h LPS-exposed *Slc37a2*^*−/−*^ macrophages exhibited reduced levels of metabolites of the purine and pyrimidine metabolism (Figure S6). These results agree with the increased PPP flux in the resting state but decreased PPP activity in LPS-exposed *Slc37a2*^*−/−*^ versus WT macrophages.

We also observed a general decrease in levels of most of the TCA cycle intermediates with a concomitant increase in phosphate (an oxidative phosphorylation end product) in *Slc37a2*^*−/−*^ macrophages at 3 h LPS stimulation, suggesting increased cycling of TCA metabolites for energy production through oxidative phosphorylation in response to LPS activation (Figures 5D and 5E). In support of this notion, we observed increased ECAR (Figures 5F and 5G) and OCR (Figures 5H and 5I) in *Slc37a2*^*−/−*^ versus WT macrophages treated with LPS for 3 h, suggesting that SLC37A2 deficiency promotes LPS-induced glycolysis and mitochondrial OXPHOS. Together, our results indicate that SLC37A2 deletion redirects glucose flux toward the glycolytic pathway and TCA cycle but limits its flux toward PPP during LPS exposure (Figure 5J).

### SLC37A2 Deficiency Promotes Cytosolic Reactive Oxidative Species Production in Macrophages

In addition to cytokine secretion, pro-inflammatory macrophages also generate reactive oxidative species (ROS), which serves as microbicidal agents in the host-defense response (Mittal et al., 2014). However, excessive ROS production at the site of inflammation also causes oxidative stress-mediated tissue damage. Mitochondrial respiration and NADPH oxidases are the two primary sources of cellular ROS in macrophages (Mittal et al., 2014). Note that PPP activity contributes to the generation of NADPH and redox balance. Given the differential activities of the PPP and mitochondrial respiration in resting and LPS-activated *Slc37a2*^*−/−*^ versus WT macrophages, we next examined macrophage redox status by measuring cellular and mitochondrial ROS using CellROX and MitoSOX, respectively. *Slc37a2*^*−/−*^ versus WT macrophages showed significantly increased cellular ROS production through CellROX staining at baseline and after 3 or 6 h of LPS stimulation (Figure 5K). No significant difference was observed in MitoSOX staining between genotypes when treated with LPS (Figure S7A) or LPS plus ATP (to induce NLRP3 inflammasome activation as a positive control) (Figure S7B). Together, our data suggest that SLC37A2 suppresses cellular, but not mitochondrial, ROS production in acute macrophage inflammation.

### Glycolysis and NAD^+^ Salvage Drive Pro-inflammation in *Slc37a2*^*−/−*^ Macrophages

**Figure 6:**
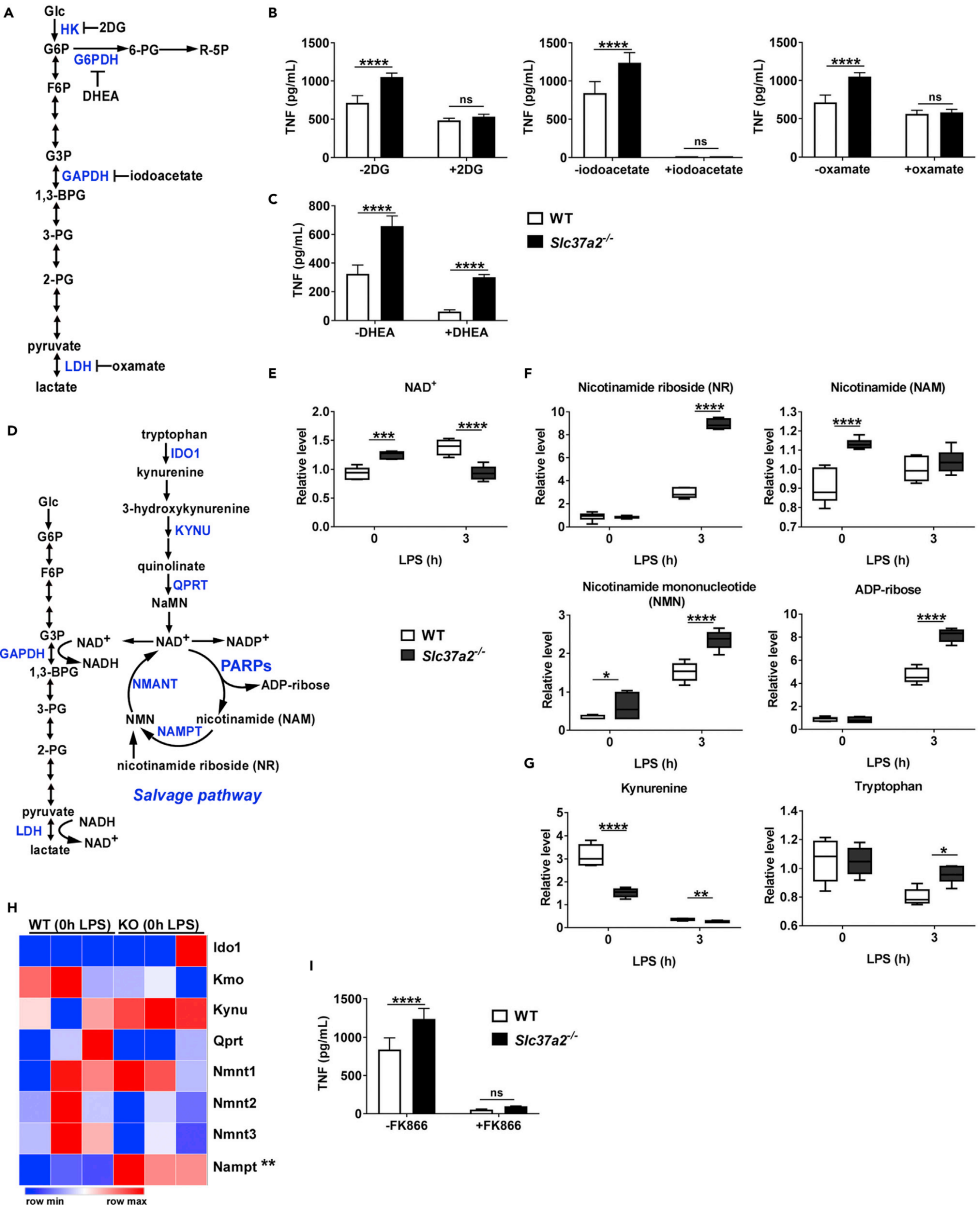
Glycolysis and NAD^+^ Salvage Drive Pro-Inflammation in *Slc37a2*^*−/−*^ Macrophages (A) Schematic representation of glycolysis. The targeting sites of the pharmaceutical inhibitors are indicated. (B and C) TNF secretion from WT and *Slc37a2*^*−/−*^ BMDMs pretreated with hexokinase inhibitor 2-deoxy-D-glucose (2-DG; 10 mM), glyceraldehyde-3-phosphate dehydrogenase (GAPDH) inhibitor iodoacetate (200 μM), lactate dehydrogenase inhibitor sodium oxamate (40 mM), glucose-6-phosphate dehydrogenase (G6PDH) inhibitor dehydroepiandrosterone (DHEA) (200 μM), followed by 100 ng/mL LPS for 6 h. (D) Schematic representation of glycolysis and NAD biosynthetic pathways. (E–G) The relative level of metabolites in NAD^+^*de novo* synthesis and salvage pathways in WT and *Slc37a2*^*−/−*^ macrophages treated with or without 100 ng/mL LPS for 3 h, assessed by unbiased metabolomics. (H) Expression of genes involved in NAD^+^*de novo* synthesis and salvage pathways in resting WT and *Slc37a2*^*−/−*^ macrophages, assessed by RNA-seq. (I) TNF secretion from WT and *Slc37a2*^*−/−*^ BMDMs pretreated with nicotinamide phosphoribosyltransferase (NAMPT) inhibitor FK866 (50 nM), followed by 100 ng/mL LPS for 6 h. Data are representative of two independent experiments with four samples per group (mean ± SEM). ∗p < 0.05, ∗∗p < 0.01, ∗∗∗p < 0.001, ∗∗∗∗p < 0.001, two-tailed Student's t test (H) or two-way ANOVA with post hoc Tukey's multiple comparisons test (B, C, E, F, G, and I).

Next, we investigated whether SLC37A2 represses macrophage pro-inflammation activation by regulating glucose metabolism. To address this question, we employed inhibitors that interfere with glycolysis and PPP and measured cytokine production in both *Slc37a2*^*−/−*^ and WT macrophages (Figure 6A). Blockade of glycolysis by 2-deoxyglucose (2DG, blocking hexokinase), iodoacetate (blocking GAPDH), and oxamate (blocking LDH) (Figure 6B), but not blockade of PPP by dehydroepiandrosterone (DHEA, blocking G6PDH) (Figure 6C), normalized the differential production of TNF between genotypes in response to 6 h of LPS stimulation, suggesting that hyper-inflammation in *Slc37a2*^*−/−*^ macrophages results from enhanced glycolytic reprogramming but not PPP flux.

Note that, during glycolysis, GAPDH activity consumes NAD^+^, whereas LDH regenerates NAD^+^. Moreover, NAD^+^-biosynthetic pathways regulate macrophage inflammation. For example, the kynurenine-mediated NAD^+^ synthesis pathway directs anti-inflammatory homeostasis (Minhas et al., 2018). In contrast, the NAD^+^ salvage pathway sustains glycolysis and inflammation by supporting NAD^+^-dependent activation of GAPDH (Cameron et al., 2019) (Figure 6D). Our metabolomics data showed that resting *Slc37a2*^*−/−*^ macrophages had increased accumulation of NAD^+^ and the metabolites in the NAD^+^ salvage pathway, including nicotinamide (NAM) and nicotinamide mononucleotide (NMN), but decreased the level of kynurenine, a metabolite in NAD^+^ *de novo* synthesis pathway (Figures 6E–6G). LPS-stimulated *Slc37a2*^*−/−*^ versus WT macrophages had significantly higher levels of nicotinamide riboside (NR), NMN, and ADP-ribose but a lower level of NAD^+^, suggesting enhanced NAD^+^ salvage activity due to rapid NAD^+^ consumption. Concomitant with increased NAD^+^ salvage, *Slc37a2*^*−/−*^ macrophages expressed a significantly higher level of nicotinamide phosphoribosyltransferase (*Nampt*), the rate-limiting enzyme in the NAD^+^ salvage pathway (Figure 6H). Furthermore, blockade of the NAD^+^ salvage pathway using FK866 (inhibits NAMPT) significantly attenuated inflammation in macrophages of both genotypes and normalized the genotypic difference in pro-inflammatory cytokine secretion (Figure 6I), suggesting that the increased inflammation in *Slc37a2*^*−/−*^ macrophages is dependent on NAD^+^ biosynthesis from the salvage pathway. As the NAD^+^ salvage pathway is required for aerobic glycolysis during acute inflammation, our results indicate that SLC37A2 deletion promotes NAD^+^ salvage-dependent glycolytic reprogramming, which is attributable to excessive inflammatory cytokine production.

### SLC37A2 Overexpression Represses Macrophage Pro-Inflammatory Activation

**Figure 7:**
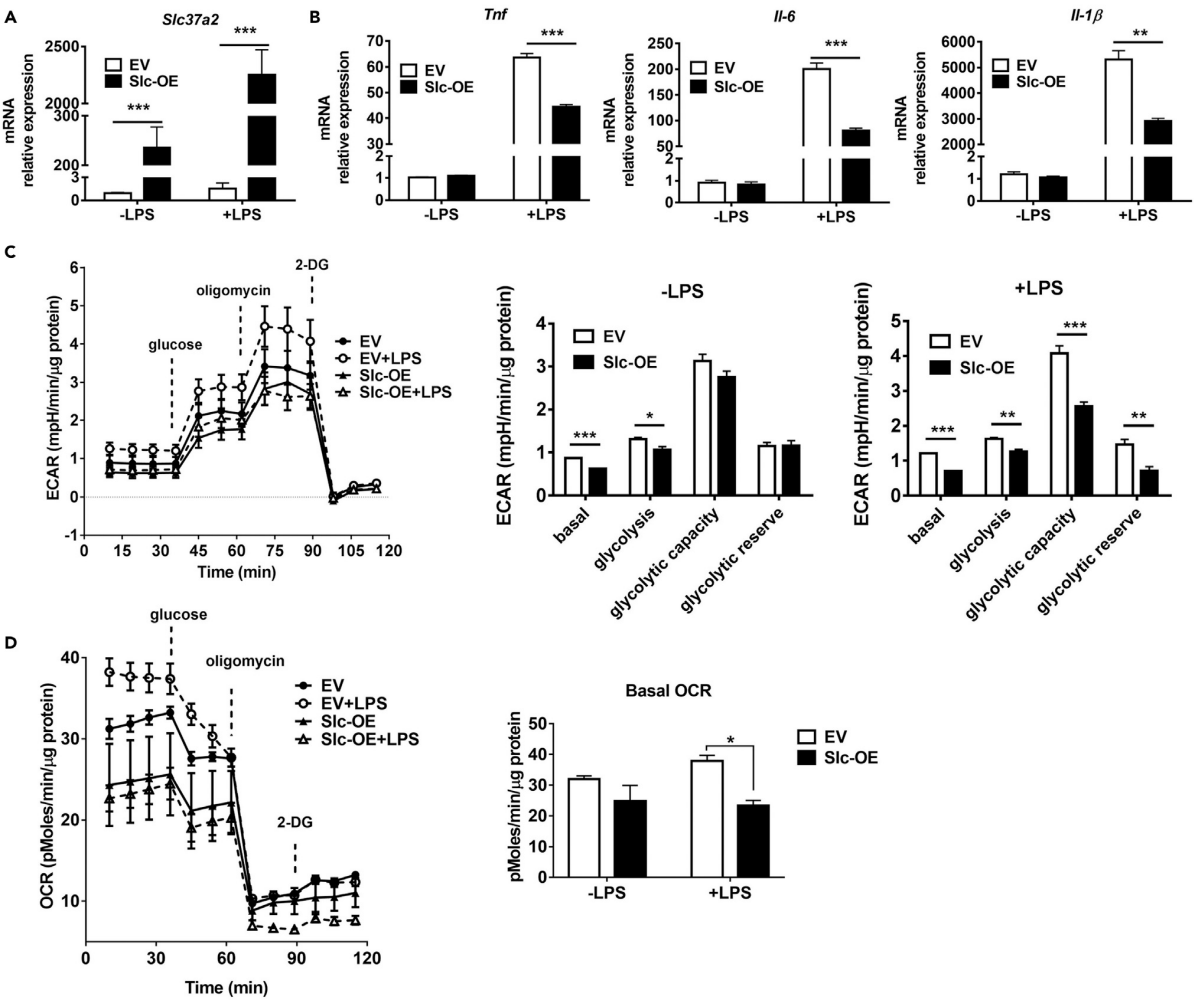
SLC37A2 Overexpression Represses Macrophage Pro-inflammatory Activation (A and B) *Slc37a2* and cytokine expression in human SLC37A2 overexpressing Raw 264.7 macrophages stably transfected with empty vector (EV) or human SLC37A2 plasmid (Slc-OE). Cells were treated with or without 100 ng/mL LPS for 4 h. (C and D) Seahorse analysis of ECAR and OCR after sequential injection of glucose (25 mM), oligomycin (1 μM), and 2-deoxyglucose (2-DG, 20 mM), in control and SLC37A2-overexpressing cells treated with or without 100 ng/mL LPS for 3 h. Data are representative of two independent experiments with four samples per group (mean ± SEM). ∗ p < 0.05, ∗∗p < 0.01, ∗∗∗ p < 0.001, two-way ANOVA with post hoc Tukey's multiple comparisons test (A and B) and unpaired, two-tailed Student's t test (C and D).

To further investigate the roles of SLC37A2 in macrophage pro-inflammatory activation, we generated a Raw264.7 stable cell line that overexpresses SLC37A2 by transfecting cells with human SLC37A2, which has 90% amino acid identity with mouse SLC37A2 (Bartoloni and Antonarakis, 2004), plasmid under the control of the CMV promoter. Human SLC37A2-overexpressing cells showed an increase in human *Slc37a2* expression at basal level and after LPS stimulation (Figure 7A), which is consistent with the induction of the CMV promoter by LPS (Ramanathan et al., 2005). As expected, SLC37A2 overexpression repressed pro-inflammatory responses, as shown by decreased *Il-1β*, *Il-6*, and *Tnf* mRNA expression in response to LPS relative to control (transfected with empty vector) cells, indicating a blunted macrophage inflammatory activation (Figure 7B). We assessed glycolysis by recording ECAR and OCR in 3 h LPS-activated SLC37A2-overexpressing and control macrophages by respirometry. SLC37A2-overexpressing macrophages showed significantly lower glycolysis and glycolytic capacity in a resting state (without LPS stimulation) and after LPS stimulation (Figure 7C). Similarly, simultaneous analysis of oxygen consumption indicates that glucose-mediated oxidative metabolism is also depressed (Figure 7D). Together, these data suggest that SLC37A2 appears to be a critical repressor of macrophage inflammatory activation by modulating cellular glucose metabolism.

## Discussion

There is a rapidly growing interest in understanding how activated macrophages reconfigure their metabolic process, which is required for proper macrophage activation to enable host defense mechanisms. Using both gain- and loss-of-function models and transcriptomics and metabolomics approaches, we identified SLC37A2 as a crucial anti-glycolytic, anti-inflammatory, and anti-oxidative stress regulator, such that upregulation of its expression serves as a negative feedback mechanism to prevent excessive inflammatory cytokine or cellular ROS production during the early phase of macrophage activation. By controlling glucose flux into aerobic glycolysis, SLC37A2 regulates the cross talk between innate immunity and cellular metabolic pathways. As a critical regulator of glucose utilization in macrophages, SLC37A2 controls early glycolytic reprogramming and balances other cellular metabolic processes during acute innate activation. Our results demonstrated that deletion of SLC37A2 drives macrophages to undergo hyper-glycolytic reprogramming in the resting state. As a consequence, SLC37A2-deficient macrophages are hyper-responsive to cell-surface and endosomal TLR stimulation. Conversely, overexpression of SLC37A2 dampens glycolysis and LPS-induced pro-inflammatory cytokine expression. As SLC37A2 protein expression is rapidly up-regulated within 1 h of LPS stimulation, our study highlights that SLC37A2 functions as an early repressor to counter against overwhelming inflammatory cytokine production and to promote resolution of inflammation during acute inflammatory activation by fine-tuning glycolytic reprogramming.

Enhanced glycolytic reprogramming has been recognized as a metabolic hallmark of pro-inflammatory macrophage activation (Kelly and O'Neill, 2015, O'Neill and Pearce, 2016, Pearce and Pearce, 2013). Upon entering the cytoplasm, glucose is rapidly converted to G6P by hexokinases. G6P can then be converted to pyruvate via glycolysis or 6-phosphogluconate via PPP. G6P can also be transported into ER via G6P transporters such as SLC37A4 (G6PT) (Chou et al., 2013). *Slc37a4*^*−/−*^ mice display neutropenia and have neutrophils with impaired chemotaxis and reduced chemokine production (Chen et al., 2003). Mechanistically, SLC37A4 functionally couples with G6Pase-β to maintain neutrophil energy homeostasis by recycling of ER glucose to the cytoplasm (Jun et al., 2010, Jun et al., 2012, Kim et al., 2008), such that SLC37A4 deficiency impairs neutrophil energy homeostasis and activation of the HIF-1α/PPARγ pathway (Jun et al., 2014). Similar to SLC37A4, SLC37A2 also functions as a Pi-linked G6P antiporter. However, SLC37A2 does not functionally couple with G6Pase-α or G6Pase-β (Pan et al., 2011). Moreover, compared with *Slcs37a4*, the *Slc37a2* transcript is highly expressed in macrophages (Pan et al., 2011, Kim et al., 2007, Chou et al., 2013), suggesting a critical role of SLC37A2 over SLC37A4 in macrophage G6P metabolism and inflammation.

Indeed, SLC37A2 deletion increased glycolytic reprogramming in resting macrophages, as evidenced by the increased level of pyruvate, increased ECAR, concomitant with increased expression of *Pfkp*, a glycolysis rate-limiting enzyme, and increased NAD^+^ salvage activity. This commitment to glycolysis in resting *Slc37a2*^*−/−*^ macrophages likely contributes to pro-inflammatory activation as LPS-induced inflammatory activation profoundly increased in *Slc37a2*^*−/−*^ macrophages. During glycolysis, GAPDH activity consumes NAD^+^, whereas LDH regenerates NAD^+^. Recent studies imply that the NAD^+^ salvage pathway supports the NAD^+^-dependent activity of GAPDH, thus sustaining inflammation (Cameron et al., 2019). We found that increased inflammation in *Slc37a2*^*−/−*^ macrophages relies on glycolysis, as glycolysis blockade at the steps of HK, GAPDH, or LDH using pharmaceutical inhibitors normalized the differential cytokine secretion between WT and *Slc37a2*^*−/−*^ macrophages. Furthermore, blockade of the NAD^+^ salvage pathway significantly attenuated and normalized pro-inflammatory cytokine secretion in WT and *Slc37a2*^*−/−*^ macrophages, suggesting that the increased inflammation in *Slc37a2*^*−/−*^ macrophages is dependent on NAD^+^ salvage. Of note, glycerol phosphate shuttle enzyme GPD2 regulates NAD^+^ cytosolic cycling and mitochondrial oxidation, driving inflammatory responses (Langston et al., 2019), indicating the complexity of the NAD^+^ regulation in macrophages. We are currently exploring the underlying mechanisms of the imbalanced NAD^+^ metabolism in *Slc37a2*^*−/−*^ macrophages.

The PI3K/Akt and Erk1/2 pathways are known to activate glycolysis and innate immunity (Papa et al., 2019, Traves et al., 2012, Huh et al., 2018, Kaneda et al., 2016, Lee et al., 2017, Han et al., 2018, Harada et al., 2009, Vadlakonda et al., 2013). The PI3K/Akt pathway regulates mTOR activity, which is a central integrator of cellular metabolism, and its signaling regulates both innate and adaptive immunity (Jones and Pearce, 2017). As the expression of many glycolytic enzymes and pro-inflammatory cytokines is controlled by the master transcriptional factor HIF-1α, which is regulated by mTORC1 (Dodd et al., 2015), we initially hypothesized that the increased inflammation in *Slc37a2*^*−/−*^ macrophages might result from increased activity of the PI3K-Akt1-mTORC1-HIF-1α pathway, a critical pathway regulating trained immunity (Cheng et al., 2014). However, blocking PI3K or mTOR did not normalize the differential cytokine expression between macrophages with or without SLC37A2 expression, indicating that despite the increased inflammatory activity in *Slc37a2*^*−/−*^ macrophages, the PI3K/Akt1/mTOR pathway does not play a detectable role in SLC37A2-mediated inflammation resolution. Interestingly, in line with the metabolically active and pro-inflammatory phenotype, basal activation of the Erk1/2 pathways was apparent in resting *Slc37a2*^*−/−*^ macrophages. Furthermore, U0126 inhibition of MEK completely normalizes the differential expression of TNF and partially normalized the differential expression of IL-6, suggesting a critical role of MEK/Erk1/2 in SLC37A2-mediated inflammation resolution.

One interesting observation in our study is that *Slc37a2*^*−/−*^ macrophages had increased Erk1/2 phosphorylation at baseline but decreased phosphorylation after 15 min of LPS stimulation, followed by a rapid increase in Erk1/2 phosphorylation at later time points. The exact mechanisms for this rapid change of Erk1/2 activation in the setting of SLC37A2 deletion are unclear. However, it is well known that Erk1/2 is activated by macrophage colony stimulating factor (M-CSF) and is essential for macrophage survival and proliferation (Bourgin-Hierle et al., 2008, Richardson et al., 2015). Moreover, pro-inflammatory stimulation suppresses proliferation and reprograms macrophage metabolism to promote rapid macrophage activation (Liu et al., 2016). Given that the resting *Slc37a2*^*−/−*^ macrophages have increased cellular ROS production, we speculate that SLC37A2 deficiency might create an oxidative stress condition in macrophages, which causes oxidative DNA damage, leading to elevated survival signaling. If true, the rapid changes of Erk1/2 phosphorylation in the *Slc37a2*^*−/−*^ macrophages might reflect a rapid shift from the survival signaling to inflammatory signaling in response to different environmental cues. As Erk1/2 has emerged as a crucial regulator of the Warburg effect (Papa et al., 2019, Traves et al., 2012, Huh et al., 2018), we are interested in investigating the cross talk between SLC37A2, glycolysis, and Erk1/2 activity in the setting of macrophage inflammation and proliferation in future studies.

Energy metabolism plays a critical role in modulating immune cell function (Ryan et al., 2019, Murphy and O'Neill, 2018). Mitochondria have emerged as central organelles that integrate metabolism and inflammatory responses. Macrophage pro-inflammatory activation increases the production of TCA cycle metabolites, including citrate, itaconate, and succinate. Citrate supports the biosynthesis of pro-inflammatory lipid mediators such as prostaglandins (Williams and O'Neill, 2018), acetylation of histones, and induction of genes encoding inflammatory mediators by providing acetyl-coA (Langston et al., 2019). Besides functioning as an immune-suppressive mediator (Mills et al., 2016, Mills et al., 2018, Cordes et al., 2016, Lampropoulou et al., 2016, Jha et al., 2015), itaconate inhibits SDH, leading to accumulation of succinate, disruption of TCA cycle, and impaired OXPHOS (Lampropoulou et al., 2016). The increased accumulation of succinate promotes ROS production and stabilizes HIF-1α, promoting glycolysis and inflammation (Mills et al., 2016, Lampropoulou et al., 2016, Tannahill et al., 2013). In our study, in addition to the increased glycolytic reprogramming, SLC37A2-deficient macrophages also reconfigure energy metabolic pathways to support a hyper-inflammatory status in macrophages. This was supported by the observations that resting *Slc37a2*^*−/−*^ macrophages synthesized less anti-inflammatory itaconate, and up-regulated oxygen consumption rate, compared with WT cells. *Slc37a2*^*−/−*^ macrophages also displayed increased glucose oxidation, as shown by increased phosphate production and oxygen consumption rate after 3 h of LPS stimulation, which likely results from increased pyruvate flux into mitochondria to fuel TCA cycle in *Slc37a2*^*−/−*^ versus WT control cells. Up-regulation of mitochondrial biogenesis, glucose oxidation, and OXPHOS by LPS has been reported in monocytes and/or macrophages during the early activation phase (Liu et al., 2015, Langston et al., 2019), which promotes the production of mitochondrial ROS, inducing oxidative stress and macrophage proinflammatory activation.

LPS-stimulated macrophages have increased PPP activity, which contributes to nucleotide synthesis by supplying precursors and generation of NADPH to support glutathione reduction, ROS production by NADPH oxidase, and fatty acid synthesis. One interesting observation in our study is that there is only a mild increase in PPP in resting *Slc37a2*^*−/−*^ versus control macrophages. Upon LPS stimulation, PPP activity decreased in *Slc37a2*^*−/−*^ macrophages, likely facilitating more G6P flux into the glycolytic pathway to support the hyper-inflammatory activation in those cells, as a result of increased PFKP and NAD^+^ salvage-supported GAPDH activity. Hence, in the absence of SLC37A2, macrophages slow down glucose flux toward PPP and redirect glucose flux primarily into the glycolytic pathway to accelerate innate immune activation (Figure 5J). As a consequence of the reduced PPP activity, SLC37A2-deficient macrophages also generate more cellular ROS, likely promoting oxidative stress.

What is the physiological significance of SLC37A2 regulation of macrophage metabolism and inflammation? When encountering infection, macrophages require a high glycolytic rate, which is essential for a fully activated inflammatory response to fight off pathogens. However, excessive cytokine production (cytokine storm) will lead to cell death and tissue/organ damage. As macrophages rapidly induce and elevate SLC37A2 protein expression upon exposure of LPS, we propose that rapid induction of SLC37A2 is an intrinsic anti-inflammatory mechanism to counter-regulate overactivation of the inflammatory process. Interestingly, *Slc37a2* mRNA was 2-fold enriched in the more metabolically active intra-abdominal versus subcutaneous fat from murine obese mice (Kim et al., 2007) and showed a 9-fold increase in ob/ob versus WT epididymal fat, indicating an association between insulin resistance, macrophage inflammation, and SLC37A2 expression. As SLC37A2 negatively regulates macrophage glycolysis and inflammation, we speculate that increased SLC37A2 expression in obesity might serve as a compensatory mechanism to lower intracellular glucose and attenuate obesity-induced inflammation. Currently, we are testing this hypothesis.

Macrophages must tightly reconfigure their metabolic and molecular signaling pathways to properly instruct their effector function. By functioning as a repressor of macrophage glycolysis and inflammation, SLC37A2 bridges signaling between nutrient/energy metabolism and innate immune activation. Our findings point to SLC37A2 as a potential target for the therapeutic manipulation of macrophage inflammation *in vivo*.

### Limitations of the Study

There are several limitations of this study. First, we did not test the role of SLC37A2 in human macrophages or extend the concept to *in vivo* animal inflammatory disease models. Confirmation of our findings in a disease model and human cells will be critical goals for the future to support the translational relevance of our findings. Second, we used only one dose of LPS (100 ng/mL) in this study. It remains unknown whether SLC37A2 will function as a repressor of macrophage inflammation under the condition of extremely low concentrations of LPS stimulation. Third, we did not explore the potential roles for SLC37A2 and SLC37A2-regulated metabolic reconfiguration in ER function and homeostasis. As discussed above, SLC37A2 functions as a Pi-linked G6P antiporter, but it does not functionally couple with ER-located G6Pase-α or G6Pase-β (Pan et al., 2011), suggesting that SLC37A2 does not regulate glucose recycling between the ER and cytosol as SLC37A4 does by coupling with G6Pase. Although the ER stress marker CHOP was not detected in SLC37A2-deficient macrophages, we cannot rule out a role for SLC37A2 in regulating ER function by controlling its compartmental metabolic homeostasis.

### Resource Availability

#### Lead Contact

Further information is available from the Lead Contact, Xuewei Zhu (xwzhu@wakehealth.edu).

#### Materials Availability

All unique reagents generated in this study are available from the Lead Contact. According to the Academic DeltaOneTM License Agreement between Wake Forest University Health Sciences (Institution or Licensee) and Deltagen Inc., the institution (Licensee) and the principal investigator (Dr. Xuewei Zhu) are not authorized to distribute, sell, sublicense, or otherwise transfer any Deltagen Materials (*Slc37a2*^*−/−*^ mice and *Slc37a2*^*−/−*^ mouse-derived materials) to any third party.

#### Data and Code Availability

Raw sequencing data were deposited in the NCBI Sequence Read Archive (SRA) under Project Accession PRJNA573732.

## Methods

All methods can be found in the accompanying Transparent Methods supplemental file.

## Supplemental Information

Document S1. Transparent Methods, Figures S1–S7, and Table S1
