# Supplementary material for: Solute Carrier Family 37 Member 2 (SLC37A2) Negatively Regulates Murine Macrophage Inflammation by Controlling Glycolysis
Source: iScience. 2020 May 4;23(5):101125. doi: 10.1016/j.isci.2020.101125 (PMC7232099; doi:10.1016/j.isci.2020.101125)
Supplement: Document S1. Transparent Methods, Figures S1–S7, and Table S1 [file mmc1.pdf]

iScience, Volume *23*

Supplemental Information

Solute Carrier Family 37 Member 2 (SLC37A2) Negatively Regulates Murine Macrophage Inflammation by Controlling Glycolysis

Zhan Wang, Qingxia Zhao, Yan Nie, Yi Yu, Biswapriya B. Misra, Manal Zabalawi, Jeff W. Chou, Chia-Chi C. Key, Anthony J. Molina, Matthew A. Quinn, Michael B. Fessler, John S. Parks, Charles E. McCall, and Xuewei Zhu

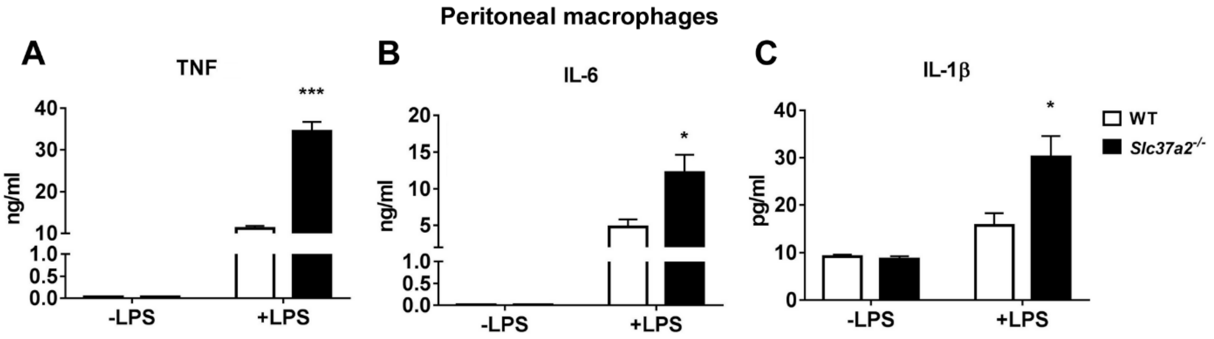

Figure S1. SLC37A2 deficiency promotes pro-inflammatory activation in murine peritoneal macrophages, Related to Figure 1. (A-C) Thioglycollate-elicited peritoneal macrophages from WT and *Slc37a2*$^{-/-}$ mice were treated with or without 100 ng/ml LPS for 6 h. Cytokine secretion was measured by ELISA. Data are representative of two independent experiments with 4 samples per group (mean ± SEM). *, P<0.05; ***; P<0.001, two-way ANOVA with post hoc Tukey's multiple comparisons test.

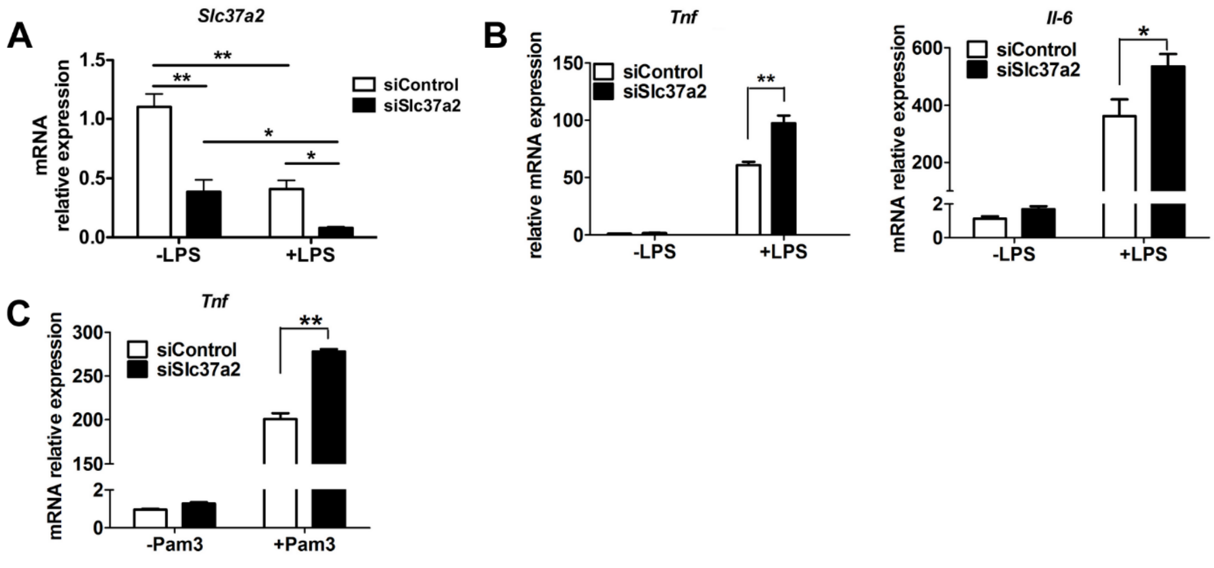

Figure S2. Silencing *Slc37a2* promotes macrophage pro-inflammatory activation, Related to Figure 1. Thioglycollate-elicited peritoneal macrophages from WT mice were transfected with control or *Slc37a2* siRNAs for 90 h before treatment ± 100 ng/ml Pam3CSK4 (TLR2 agonist) or 100 ng/ml LPS for 6 h. (**A-B**) *Slc37a2*, *Tnf*, and *Il-6* mRNA expression in control siRNA and *Slc37a2* siRNA transfected macrophages treated with or without LPS. (**C**) *Tnf* mRNA expression in control and *Slc37a2* siRNA transfected macrophages treated with or without Pam3CSK4 (Pam3). Data are representative of 2 independent experiments with 3 samples per group (mean ± SEM). *, P<0.05; **; P<0.01, two-way ANOVA with post hoc Tukey's multiple comparisons test.

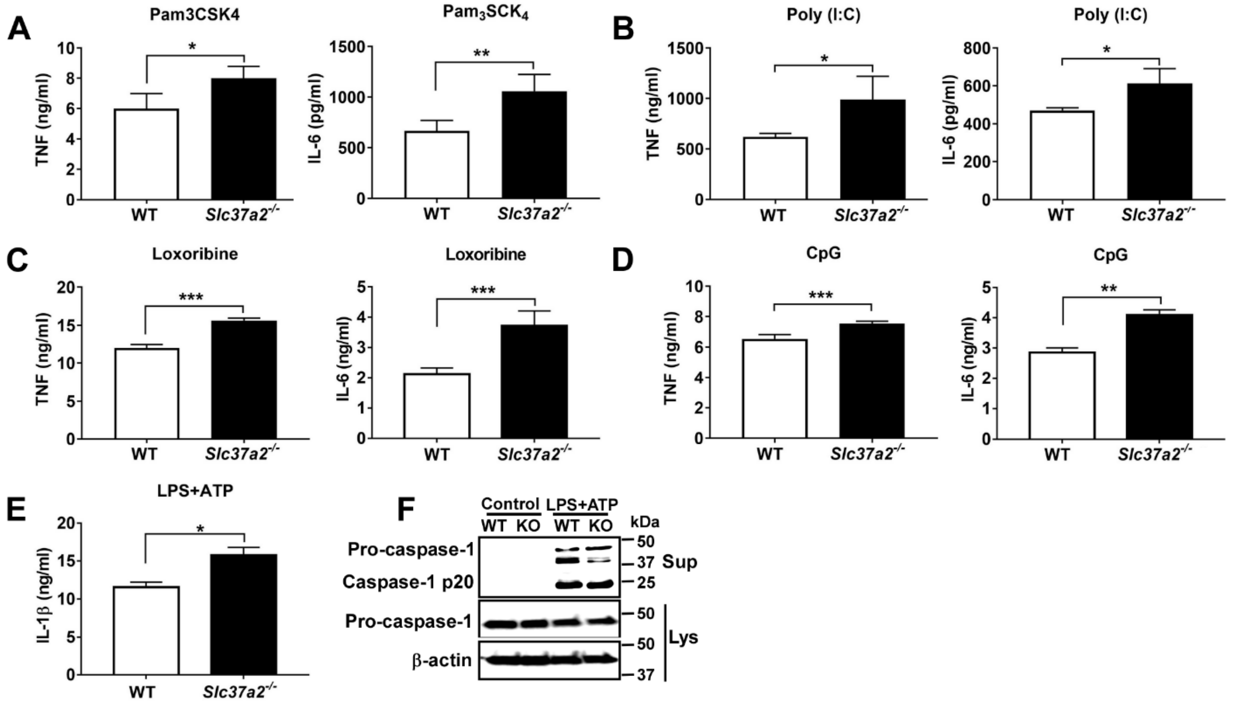

Figure S3. SLC37A2 deficiency enhances cell-surface and endosomal TLR activation but has no effect on ATP-induced caspase-1 cleavage in macrophages, Related to Figure 1. **(A-D)** Bone marrow-derived macrophages (BMDMs) from WT and *Slc37a2*$^{-/-}$ mice were treated with or without 100 ng/ml Pam3CSK4, 10 µg/ml poly (I:C), 200 µM Loxoribine, or 1 µM CpG for 8 h to activate TLR2, TLR3, TLR7, and TLR9, respectively. Cytokine release in response to TLR stimulation was measured by ELISA. (**E-F**) BMDMs from WT and *Slc37a2*$^{-/-}$ mice were treated with or without 300 ng/ml LPS for 3 h, followed by 5 mM ATP for 1 h to induce NLRP3 inflammasome activation. IL-1β secretion (**E**) and caspase-1 cleavage (**F**) in response to ATP stimulation were assayed using ELISA or immunoblotting, respectively. Sup: supernatant; Lys: cell lysates. Data are representative of 3 independent experiments with 4 samples per group (mean ± SEM). *, P<0.05; **, P<0.01; ***; P<0.001; unpaired, two-tailed Student's t-test.

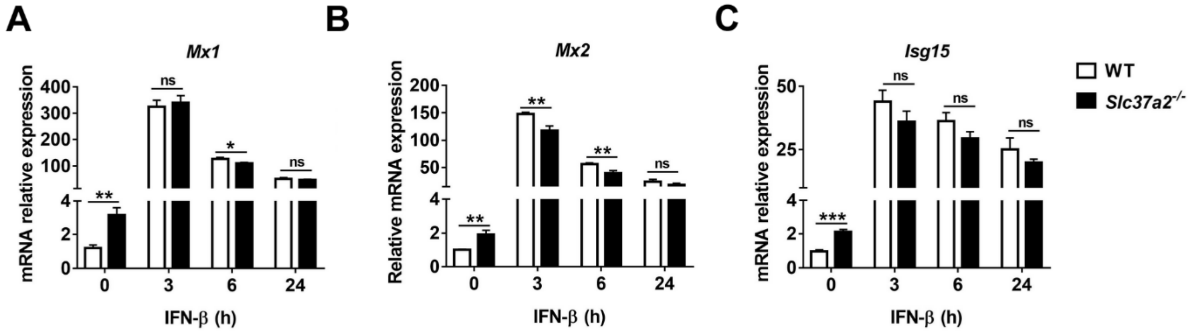

Figure S4. SLC37A2 deficiency enhances basal interferon (IFN)-β signaling activation in resting macrophages, Related to Figure 1. Bone marrow-derived macrophages (BMDMs) from WT and *Slc37a2*$^{-/-}$ mice were treated with 25 ng/ml murine IFN-β for 0, 3, 6, and 24 h. Transcript expression levels of IFN-stimulated genes, including *Mx1* (**A**), *Mx2* (**B**), and IFN-stimulated gene 15 (*Isg15*) (**C**), were measured by qPCR. Data are representative of 2 independent experiments with 4 samples per group (mean ± SEM). *, P<0.05; **, P<0.01; ***; P<0.001; ns, not significant; two-way ANOVA with post hoc Tukey's multiple comparisons test.

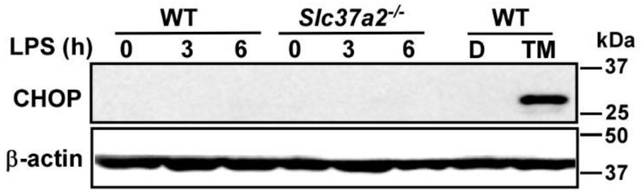

Figure S5. SLC37A2 deficiency does not induce ER stress, Related to Figure 2. Bone marrow-derived macrophages (BMDMs) from WT and *Slc37a2*$^{-/-}$ mice were treated 100 ng/ml LPS for 0-6 h to activate TLR4. WT BMDMs were treated with 10 μg/ml tunicamycin (TM) for 10 h to induce ER stress as a positive control. DMSO (D) was used as vehicle control. The ER stress protein CHOP was analyzed by Western blotting. β-actin was used as a loading control.

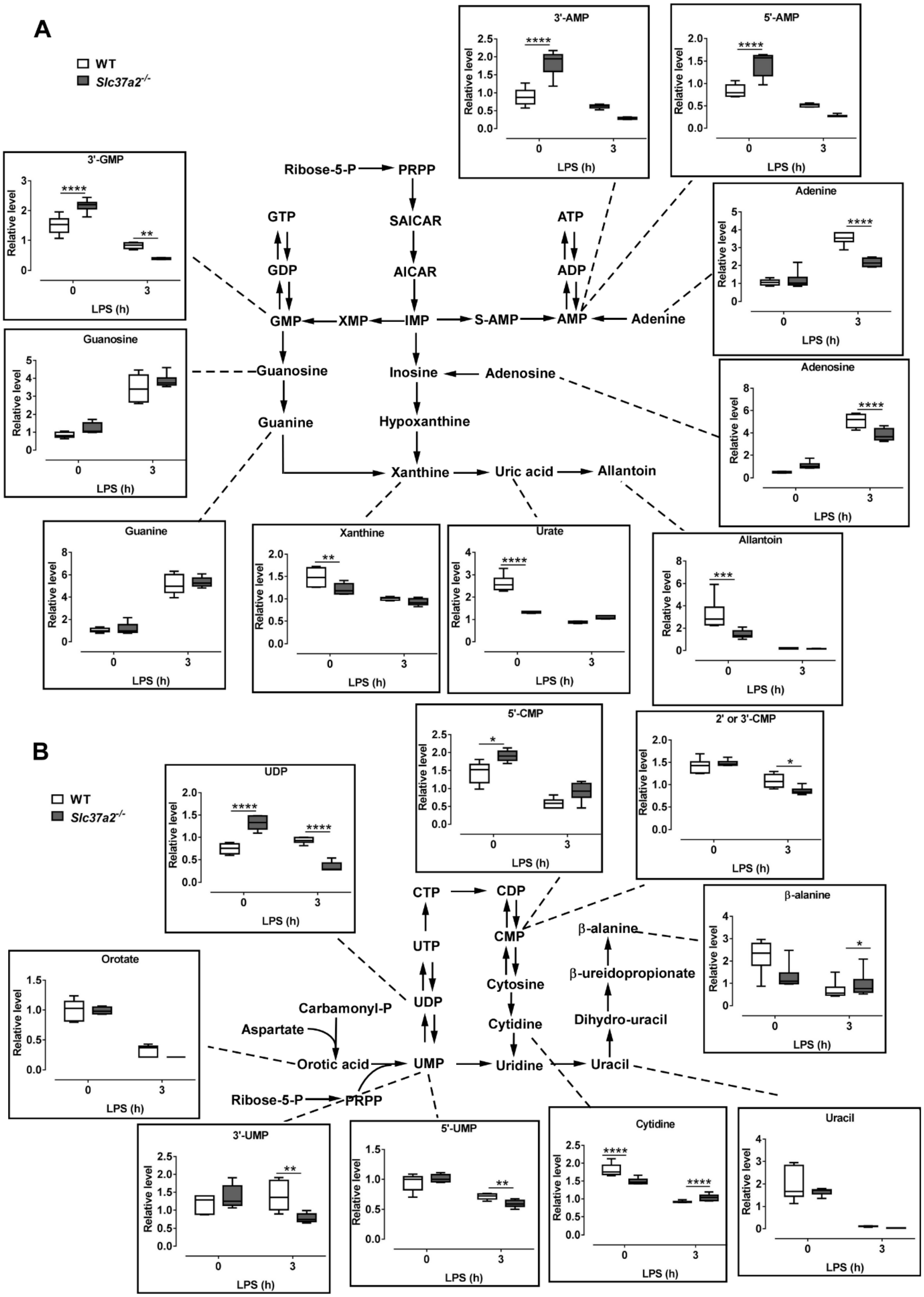
A
WT
Slc37a2-/-
3'-AMP
5'-AMP
3'-GMP
Adenine
Guanosine
Adenosine
Guanine
Xanthine
Urate
Allantoin
Relative level
LPS (h)
Ribose-5-P
PRPP
SAICAR
AICAR
GTP
GDP
GMP
XMP
IMP
S-AMP
ATP
ADP
AMP
Inosine
Hypoxanthine
Uric acid
B
5'-CMP
2' or 3'-CMP
UDP
β-alanine
Orotate
3'-UMP
5'-UMP
Cytidine
Uracil
CTP
CDP
CMP
UTP
UMP
Cytosine
Uridine
β-ureidopropionate
Dihydro-uracil
Carbamonyl-P
Aspartate
Orotic acid

Figure S6. SLC37A2 deficiency alters purine and pyrimidine metabolism, Related to Figures 4 and 5. The relative level of metabolites in purine and pyrimidine synthesis pathways in WT and *Slc37a2*$^{-/-}$ BMDMs treated with or without 100 ng/ml LPS, assessed by unbiased metabolomics. Data are represented as mean ± SEM (n=6 technical replicates). *P< 0.05, **P< 0.01, ***P<0.001, ****P<0.0001; two-way ANOVA with post doc Tukey's multiple comparisons test.

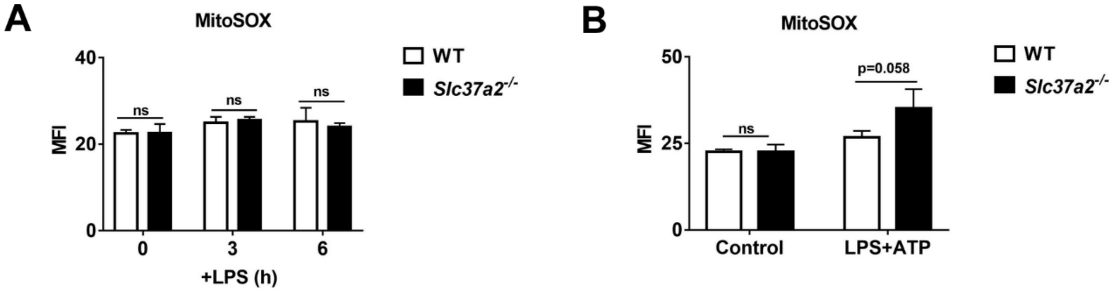

Figure S7. SLC37A2 deficiency does not increase mitochondrial reactive oxidative species (ROS) production in macrophages, Related to Figure 5. (**A**) Bone marrow-derived macrophages (BMDMs) from WT and *Slc37a2*$^{-/-}$ mice were treated with or without 100 ng/ml LPS for 0-6 h. Cells were stained with 5 µM MitoSOX for 15 min and then analyzed by flow cytometry. (**B**) BMDMs from WT and *Slc37a2*$^{-/-}$ mice were treated with or without 300 ng/ml LPS for 3 h, followed by 5 mM ATP for 1 h to induce NLRP3 inflammasome activation. Cells were stained with 5 µM MitoSOX for 15 min and then analyzed by flow cytometry. Data are representative of 3 independent experiments with 4 samples per group (mean ± SEM). ns, not significant; two-way ANOVA with post hoc Tukey's multiple comparisons test.

Table S1. Forward and reverse primers used in qPCR, Related to Figures 1 and 7

Gene name	Forward primer (5'→3')	Reverse primer (5'→3')
Gapdh	TGTGTCCGTCGTGGATCTGA	CCTGCTTCACCACCTTCTTGAT
Isg15	GGTGTCCGTGACTAACTCCAT	TGGAAAGGGTAAGACCGTCCT
Il-1β	GTCACAAGAAACCATGGCACAT	GCCCATCAGAGGCAAGGA
Il-6	CTGCAAGAGACTTCCATCCAGTT	AGGGAAGGCCGTGGTTGT
Mcp-1	TTC CTCCACCACCATGCAG	CCAGCCGGCAACTGTGA
Mx1	GACCATAGGGGTCTTGACCAA	AGACTTGCTCTTTCTGAAAAGCC
Mx2	GAGGCTCTTCAGAATGAGCAAA	CTCTGCGGTCAGTCTCTCT
Slc37a2 (human)	GTACCGGTCAGGCAAAATG	GCAGACGGCTCTTGACGATA
Slc37a2 (mouse)	GCCTGCGGCAGAAGCAGTGG	AGCAGGGGTGGCCCATGTTG
Tnf	GGCTGCCCCGACTACGT	ACTTTCTCCTGGTATGAGATAGCAAAT

Transparent Methods

Animals

Slc37a2 global knockout (KO) mice in the C57BL/6J background (T1837) were purchased from Deltagen, Inc (San Mateo, CA). Heterozygous *Slc37a2KO* mice were intercrossed to obtain wild type (WT) and homozygous KO (*Slc37a2*$^{-/-}$) mice. Mice were housed in a pathogen-free facility on a 12 h light/dark cycle and received a standard laboratory chow diet. All experimental protocols were approved by the Wake Forest University's Institutional Animal Care and Use Committee.

Macrophage culture and inflammatory stimulation

Bone marrow-derived macrophages (BMDMs) and elicited peritoneal macrophages were cultured as described previously (Zhu et al., 2008). As we did not observe significant effects of sex on inflammatory gene expression between WT and *Slc37a2*$^{-/-}$ mouse macrophages, both genders of mice were used for macrophage culture in this study. Briefly, bone marrow was isolated from male or female mice between 12 and 20 weeks of age and cultured in low glucose Dulbecco's modified Eagle's medium (DMEM) containing 30% L929 cell-conditioned medium, 20% fetal bovine serum (FBS), 2 mM L-glutamine, 1 mM sodium pyruvate, 100 U/ml penicillin, and 100 µg/ml streptomycin for 6-7 days until the cells reached confluence. BMDMs were then lifted from the dishes with 1x Versene solution (Thermo Fisher Scientific, Waltham, MA) and incubated overnight in RPMI 1640 medium containing 1% Nutridoma-SP medium (Sigma-Aldrich, St. Louis, MO). Elicited peritoneal macrophages were collected 3 days after injection of 1 ml of 10% thioglycollate into the peritoneal cavity of 12-20 weeks old male or female mice. The peritoneal cells were plated in RPMI-1640 media containing 10% FBS, 100 U/ml penicillin, and 100 µg/ml streptomycin. After a 2 h incubation, floating cells were removed by washing with phosphate-buffered saline (PBS), and adherent macrophages were used for experiments. Macrophages were treated with 100 ng/ml LPS (E. coli 0111; B4, Sigma-Aldrich, St. Louis, MO), 100 ng/ml Pam3SCK4 (InvivoGen, San Diego, CA), 10 µg/ml poly (I: C) (InvivoGen, San Diego, CA), 200 µM Loxoribine (InvivoGen, San Diego, CA), or 1 µM CpG (InvivoGen, San Diego, CA) for various times (defined in figure legends) in DMEM medium containing 25 mM glucose or RPMI-1640 medium. In some experiments, BMDMs were pretreated for 30 min to 1 h with IκB inhibitor Bay 11-7082 (5 µM; Calbiochem, San Diego, CA), MEK/ERK1/2 inhibitor U0126 (25 µM; Calbiochem, San Diego, CA), p38 inhibitor (p38 MAPK inhibitor III; 2 µM; Calbiochem, San Diego, CA), PI3K inhibitor Ly294002 (20 µM, Calbiochem, San Diego, CA), mTOR inhibitor rapamycin (50 nM, Sigma-Aldrich, St. Louis, MO), mTOR inhibitor Torin 1 (100 nM, Tocris Bioscience, Minneapolis, MN), hexokinase inhibitor 2-deoxy-D-glucose (2-DG; 10 mM, Sigma-Aldrich, St. Louis, MO), glyceraldehyde-3-phosphate

dehydrogenase (GAPDH) inhibitor sodium iodoacetate (200 μM, Sigma-Aldrich, St. Louis, MO), lactate dehydrogenase inhibitor sodium oxamate (40 mM, Santa Cruz Biotechnology, Inc, Dallas, TX), glucose-6-phosphate dehydrogenase (G6PDH) inhibitor DHEA (200 μM, EMD Millipore, Burlington, MA), and nicotinamide phosphoribosyltransferase (NAMPT) inhibitor FK866 (50 nM, Cayman Chemical, Ann Arbor, MI), and subsequently treated with 100 ng/mL LPS for an additional 6 h in the presence of each inhibitor. PBS, DMSO, or ethanol were used as vehicle control in the inhibitor experiments. In separate experiments, BMDMs were treated with 10 μg/ml tunicamycin (TM, Cayman Chemical, Ann Arbor, MI) for 0-10 h to induce ER stress, or first primed with 300 ng/ml LPS for 3 h, followed by 5 mM ATP (Sigma-Aldrich, St. Louis, MO) for 1 h to induce NLRP3 inflammasome activation, or treated with 25 ng/ml murine interferon (IFN)-β (Sino Biological, Wayne, PA) for 0-24 h to activate the IFN-β receptor pathway.

Gene expression analysis by quantitative PCR (qPCR) and ELISA

qPCR: Total RNA was isolated from macrophages using TRIzol reagent (Thermo Fisher Scientific, Waltham, MA). Single-strand cDNA was synthesized with Omniscript RT Kit (Qiagen, Germantown, MD). The relative mRNA expression level of each target gene was quantified by qPCR. Data were normalized to glyceraldehyde-3-phosphate dehydrogenase (GAPDH) and expressed relative to WT macrophages without LPS treatment using the $2^{-\Delta\Delta}$ CT method (Livak and Schmittgen, 2001). The sequences of the primers are listed in **Table S1**.

ELISA: The culture supernatants were collected and stored at -80 °C for mouse IL-1β, TNF, and IL-6 cytokine ELISA (BD Bioscience, San Jose, CA) according to the manufacturer's instructions.

Western blotting

For immunoblotting, BMDMs were lysed in radioimmunoprecipitation assay (RIPA) buffer containing protease inhibitor cocktails and PhosSTOP (Roche). Protein concentration was measured using the BCA Protein Assay Kit (Pierce). Cell proteins (10 µg) were separated on 12% SDS-polyacrylamide gels, transferred to PVDF members, and incubated overnight at 4 °C with the primary antibodies depending on the experiment. Primary antibodies used were anti-p-Akt (S473), anti-p-Akt (T308), anti-Akt, anti-p-Erk1/2, anti-Erk1/2, anti-p-p38, anti-p38, anti-p-JNK, anti-JNK, anti-IκB-α, anti-mTOR, anti-p-mTOR (S2448), anti-p-p70S6 (S371), anti-p-p70S6 (T389), anti-p-4E-BP1 (T37/46), anti-TSC2, anti-p-TSC2 (T1462), anti-p-PRAS40, anti-PRAS40, anti-p-GSK3β, anti-GSK3β, anti-CHOP (all purchased from Cell Signaling Technology, Danvers, MA and used at a dilution of 1:1000), anti-caspase-1 p20 (AdipoGen, San Diego, CA, 1:1000),

and anti-β-actin (Sigma-Aldrich, St. Louis, MO, 1:6000). Rabbit anti-SLC37A2 polyclonal antibody was made against the peptide CTPPRHHDDPEKEQDNPEDPVNSPYSSRES (LAMPIRE Biological Lab Inc., Everett, PA) and used at a dilution of 1:500. The proteins were detected with a peroxidase-linked antibody to rabbit or mouse IgG (GE Healthcare Life Sciences, Pittsburgh, PA, 1:5000, or Cell Signaling Technology, Danvers, MA, 1:2000). Immunoblots were visualized with the Supersignal substrate system (Thermo Fisher Scientific, Waltham, MA), and chemiluminescence was captured with an LAS-3000 imaging system (Fujifilm Life Science, Cambridge, MA). The intensity of the protein bands was quantified using NIH ImageJ software.

SLC37A2 overexpression cell line

RAW264.7 mouse macrophages (ATCC TIB-71) were transfected with either the empty vector (pCMV6-SLC37A2-Myc-DDK tagged cloning vector, cat#PS100016, Origene Biotechnology, Rockville, MD) or hSLC37A2 construct (pCMV6-SLC37A2-Myc-DDK, Origene Biotechnology, Rockville, MD) using Fugene HD transfection reagent (Promega, Madison, WI). Stable cell lines were established by culturing cells in a selection medium containing 400 μg/ml of neomycin/G418 (Invitrogen, Carlsbad, CA). The stable expression lines were maintained in a low stringency selection medium containing 100 μg/ml of neomycin/G418.

siRNA transfection

50 nM control or Slc37a2siRNA (Dharmacon, Lafayette, CO) was transfected into elicited peritoneal macrophages or RaW264.7 macrophages with DharmaFECT 1 transfection reagent (Dharmacon, Lafayette, CO) according to the manufacturer's protocol. To evaluate silencing efficiency, qPCR of cellular RNA collected 48 h after transfection was performed. siRNA-treated macrophages were then incubated for 4 h with or without 100 ng/ml LPS or TLR2 agonist Pam3csk4 (Invivogen, San Diego, CA) before analysis of cytokine expression by qPCR.

Untargeted metabolomics analysis

Sample preparation: PBS-washed BMDMs were detached by scraping and pelleted for untargeted metabolomics analysis by Metabolon, Inc. (Durham, NC) as described (Zhu et al., 2019). Briefly, samples were prepared using the automated MicroLab STAR® system from Hamilton Company. Several recovery standards were added before the first step in the extraction process for purposes of quality control. After removal of protein, the sample extract was divided into five fractions: one for analysis by ultrahigh performance liquid chromatography-tandem mass spectroscopy (UPLC-MS/MS) with positive ion mode electrospray ionization, one for analysis by

UPLC-MS/MS with negative ion mode electrospray ionization, one for analysis by UPLC-MS/MS polar platform (negative ionization), one for analysis by GC-MS, and one sample was saved for backup.

UPLC-MS/MS: All methods utilized a Waters ACQUITY ultra-performance liquid chromatography (UPLC) and a Thermo Scientific Q-Exactive high resolution/accurate mass spectrometer interfaced with a heated electrospray ionization (HESI-II) source and Orbitrap mass analyzer operated at 35,000 mass resolution, as described (Zhu et al., 2019).

Data extraction and compound Identification: Raw data was extracted, peaks were identified, and quality control was processed using Metabolon's software. Compounds were identified by comparison to library entries of purified standards or recurrent unknown entities. Biochemical identifications are based on three criteria: retention index within a narrow RI window of the proposed identification, accurate mass match to the library +/- 10 ppm, and the MS/MS forward and reverse scores between the experimental data and authentic standards. The MS/MS scores are based on a comparison of the ions present in the experimental spectrum to the ions present in the library spectrum. A variety of curation procedures were carried out to ensure that a high-quality data set was made available for statistical analysis and data interpretation.

Data analysis: Data are presented as relative measures of "scaled intensity" after normalization to protein and median scaling to 1. The KEGG (Kyoto Encyclopedia of Genes and Genomes) pathway enrichment, unsupervised principal component analysis, and random forest analysis were performed using MetaboAnalyst (www.metaboanalyst.ca).

Seahorse assay

2×10^5 BMDMs or 1×10^5 Raw264.7 macrophages were plated into each well of Seahorse X24 cell culture microplates (Agilent Technologies, Santa Clara, CA) and cultured overnight before treated with or without 100 ng/ml LPS for 3 h. Basal and LPS-induced changes in oxygen consumption rate (OCR) and extracellular acidification (ECAR) rates in BMDMs were measured with a Seahorse XF24 Extracellular Flux Analyzer (Agilent Technologies, Santa Clara, CA) under basal conditions and following the sequential addition of 25 mM glucose, 1 µM oligomycin, 1.5 µM fluoro-carbonyl cyanide phenylhydrazone (FCCP), and 100 nM rotenone plus 1 µM antimycin A (all the compounds were from Agilent Technologies, Santa Clara, CA). OCR and ECAR in Raw 264.7 cells were measured under the same conditions except with the sequential addition of 25

mM glucose, 1 µM oligomycin, and 20mM 2DG. Results were collected with Wave software version 2.6 (Agilent Technologies, Santa Clara, CA).

Lactate assay

The concentrations of lactate in the cells and medium were measured with a Lactate Assay Kit (Biovision Inc., Milpitas, CA) according to the manufacturer's instructions.

Reactive oxidative species (ROS) measurement by flow cytometry

BMDMs were treated with 100 ng/ml LPS for 0-6 h or 300 ng/ml LPS for 3 h, followed by 5 mM ATP for 1 h before staining. For mitochondrial ROS staining, BMDMs were incubated with 5 µM MitoSOX (Thermo Fisher Scientific, Waltham, MA) in HBSS plus calcium and magnesium (Sigma) for 15 min at 37°C. For cellular ROS staining, BMDMs were incubated with 5 µM CellROX deep red (Thermo Fisher Scientific, Waltham, MA) for 30 min at 37°C. After staining, BMDMs were washed three times and gently lifted from the tissue culture plates. Data were acquired by flow cytometry on a FACSCalibur or LSRFortessa (BD Biosciences, San Jose, CA) and analyzed with FlowJo v.10.1 (FlowJo, LLC).

RNA sequencing (RNAseq)

RNA preparation and sequencing: BMDMs were lysed in Buffer RLT (Qiagen, Germantown, MD) plus β-mercaptoethanol. RNA was extracted using the Qiagen AllPrep DNA/RNA kit (Qiagen, Germantown, MD) and processed using the standard manual protocol. RNA was DNase-treated and purified using the RNA Clean and Concentrator-5 kit (Zymo Research, Irvine, CA) and assessed for RNA quality using an Agilent 2100 Bioanalyzer and the RNA 6000 Nano Kit (Agilent Technologies, Santa Clara, CA). Total RNA was used to prepare cDNA libraries using the Illumina® TruSeq Stranded Total RNA with the Ribo-Zero Gold Preparation kit (Illumina Inc., San Diego, CA). The RNA quality was excellent with RIN values ranging from 9.4 to 10. Briefly, 750 ng of total RNA was rRNA depleted, followed by enzymatic fragmentation, reverse-transcription, and double-stranded cDNA purification using AMPure XP magnetic beads. The cDNA was end-repaired, 3′ adenylated, with Illumina sequencing adaptors ligated onto the fragment ends, and the stranded libraries were pre-amplified with PCR. The library size distribution was validated and quality inspected using a Fragment Analyzer (Advanced Analytical Solutions, Parkersburg, WV). The quantity of each cDNA library was measured using the Qubit 3.0 (Thermo Fisher Scientific, Waltham, MA). The libraries were pooled and sequenced to a target read depth of 40M reads per

library using single-end 76 cycle sequencing with the High Output 75-cycle kit (Illumina Inc., San Diego, CA) on the Illumina NextSeq 500.

RNAseq data analysis: Alignment of reads was performed using the STAR sequence aligner 2.5.1 (Dobin et al., 2013), and then the gene expression in counts was extracted using FeatureCounts 1.6.1 (Liao et al., 2014). Differentially expressed genes (DEGs) were analyzed using DESeq2 1.4.5 (Love et al., 2014), where gene counts were used as inputs. Significant DEGs were conservatively defined as $p<0.05$ after adjustment for false discovery rate and fold change > 1.5 or < 0.5 between the two samples of biological replicates. In addition, DEGs were analyzed using DAVID 6.8 (Huang et al., 2007) or Qlucore Omics Explorer 3.4 (Qlucore, New York, NY) to identify enriched biological ontology. Heat maps were generated using Qlucore Omics Explorer 3.4 (Qlucore, New York, NY) or Morpheus (Broad Institute, Cambridge, MA).

Statistical analysis

Statistical analysis was performed using GraphPad Prism software 7 (GraphPad Software, San Diego, CA). Data are presented as the mean ± SEM unless indicated otherwise. Comparisons of two groups were calculated using unpaired two-tailed Student's t-tests; comparisons of more than two groups were calculated using two-way analysis of variance (ANOVA) with post hoc Tukey's multiple comparisons test. P values and statistical analysis are indicated in the figure legends. Normal distribution was observed between groups in individual comparisons. The selection of sample size was based on our experience with macrophage cytokine expression and metabolic assays.

Supplemental References

DOBIN, A., DAVIS, C. A., SCHLESINGER, F., DRENKOW, J., ZALESKI, C., JHA, S., BATUT, P., CHAISSON, M. & GINGERAS, T. R. 2013. STAR: ultrafast universal RNA-seq aligner. Bioinformatics, 29, 15-21.

HUANG, D. W., SHERMAN, B. T., TAN, Q., KIR, J., LIU, D., BRYANT, D., GUO, Y., STEPHENS, R., BASELER, M. W., LANE, H. C. & LEMPICKI, R. A. 2007. DAVID Bioinformatics Resources: expanded annotation database and novel algorithms to better extract biology from large gene lists. Nucleic Acids Res, 35, W169-75.

LIAO, Y., SMYTH, G. K. & SHI, W. 2014. featureCounts: an efficient general purpose program for assigning sequence reads to genomic features. Bioinformatics, 30, 923-30.

LIVAK, K. J. & SCHMITTGEN, T. D. 2001. Analysis of relative gene expression data using real-time quantitative PCR and the 2(-Delta Delta C(T)) Method. Methods, 25, 402-8.

LOVE, M. I., HUBER, W. & ANDERS, S. 2014. Moderated estimation of fold change and dispersion for RNA-seq data with DESeq2. Genome Biol, 15, 550.

ZHU, X., LEE, J. Y., TIMMINS, J. M., BROWN, J. M., BOUDYGUINA, E., MULYA, A., GEBRE, A. K., WILLINGHAM, M. C., HILTBOLD, E. M., MISHRA, N., MAEDA, N. & PARKS, J. S. 2008. Increased cellular free cholesterol in macrophage-specific Abca1 knock-out mice enhances pro-inflammatory response of macrophages. J Biol Chem, 283, 22930-41.

ZHU, X., MEYERS, A., LONG, D., INGRAM, B., LIU, T., YOZA, B. K., VACHHARAJANI, V. & MCCALL, C. E. 2019. Frontline Science: Monocytes sequentially rewire metabolism and bioenergetics during an acute inflammatory response. J Leukoc Biol, 105, 215-228.
